# Genome-Wide Identification and Characterization of bHLH Transcription Factors Related to Anthocyanin Biosynthesis in Red Walnut (*Juglans regia* L.)

**DOI:** 10.3389/fgene.2021.632509

**Published:** 2021-02-24

**Authors:** Wei Zhao, Yonghui Liu, Lin Li, Haijun Meng, Ying Yang, Zhaobin Dong, Lei Wang, Guoliang Wu

**Affiliations:** ^1^College of Horticulture, Henan Agricultural University, Zhengzhou, China; ^2^Henan Key Laboratory of Fruit and Cucurbit Biology, Zhengzhou, China; ^3^Shangluo Shengda Industrial Co., Ltd., Luonan, China

**Keywords:** red walnut, bHLH transcription factors, anthocyanin biosynthesis, genome-wide identification, gene expression

## Abstract

Basic helix-loop-helix (bHLH) proteins are transcription factors (TFs) that have been shown to regulate anthocyanin biosynthesis in many plant species. However, the *bHLH* gene family in walnut (*Juglans regia* L.) has not yet been reported. In this study, 102 *bHLH* genes were identified in the walnut genome and were classified into 15 subfamilies according to sequence similarity and phylogenetic relationships. The gene structure, conserved domains, and chromosome location of the genes were analyzed by bioinformatic methods. Gene duplication analyses revealed that 42 *JrbHLHs* were involved in the expansion of the walnut *bHLH* gene family. We also characterized *cis*-regulatory elements of these genes and performed Gene Ontology enrichment analysis of gene functions, and examined protein-protein interactions. Four candidate genes (*JrEGL1a*, *JrEGL1b*, *JrbHLHA1*, and *JrbHLHA2*) were found to have high homology to genes encoding bHLH TFs involved in anthocyanin biosynthesis in other plants. RNA sequencing revealed tissue- and developmental stage-specific expression profiles and distinct expression patterns of *JrbHLHs* according to phenotype (red vs. green leaves) and developmental stage in red walnut hybrid progeny, which were confirmed by quantitative real-time PCR analysis. All four of the candidate JrbHLH proteins localized to the nucleus, consistent with a TF function. These results provide a basis for the functional characterization of *bHLH* genes and investigations on the molecular mechanisms of anthocyanin biosynthesis in red walnut.

## Introduction

Walnut (*Juglans regia* L.) is among the top four economically and ecologically important nuts worldwide (Zheng et al., 2020). Walnuts have been praised as the “superfood of the 21st century” because of their abundance of nutrients, such as unsaturated fatty acids, proteins, sugars, cellulose, vitamins, and minerals (Li et al., 2018a). The leaf and pericarp of the most widely cultivated walnut varieties are green and the seed coat is generally light yellow. The red walnut found in China has a red leaf, pericarp, seed coat, and xylem (Supplementary Figure S1; Li et al., 2018b). The leaf and pericarp have been shown to contain a large number of anthocyanins (Wang et al., 2009; Li et al., 2018a), which are secondary metabolites that are usually found in the flower, fruit, leaf, stem, and root of plants and play critical roles in pigmentation, fruit quality, and pathogen resistance (Ji et al., 2019). However, the molecular mechanisms of pigment formation in walnut have not yet been elucidated.

Anthocyanin biosynthesis is affected by intrinsic and environmental factors and involves enzymatic reactions and modulation of regulatory factors (Supplementary Figure S2; Misyura et al., 2013). Key enzymes encoded by structural genes have been shown to promote anthocyanin biosynthesis, including chalcone synthase, chalcone isomerase, dihydroflavonol 4-reductase, and anthocyanidin synthase; their spatial and temporal expression patterns are regulated by various transcription factors (TFs; Butelli et al., 2012). The functions of structural genes are for the most part conserved, and interspecies differences in the mechanisms of anthocyanin accumulation are attributable to the activities of distinct TFs.

bHLH proteins constitute the second largest superfamily of TFs and play an important role in anthocyanin biosynthesis. The bHLH motif contains ~60 amino acid residues and includes 2 functional domains: the C-terminal HLH region and N-terminal basic amino acid region (which interacts with *cis*-regulatory elements; Toledo-Ortiz et al., 2003). The HLH loop has a variable length and can form homo- or heterodimers depending on the interactions of hydrophobic amino acids (Li et al., 2006). In general, the conserved motifs of bHLH proteins are involved in protein-protein interactions (PPIs; Feller et al., 2017). bHLH TFs regulate many physiologic processes and metabolic pathways in plants including anthocyanin biosynthesis (Hu et al., 2016; Zhao et al., 2017). The first bHLH TF identified in plants was the Lc protein encoded by the R gene in maize, which was shown to regulate at least 2 structural genes involved in anthocyanin biosynthesis (Chandler et al., 1989). Other bHLH TFs related to anthocyanin biosynthesis that have been identified in plants include *AtEGL3*, *AtGL3*, and *AtTT8* in *Arabidopsis* (Bailey et al., 2003); *VvMYCA1* in grape (*Vitis vinifera*; Matus et al., 2010); *NtAn1* and *NtAn2* in tobacco (*Nicotiana tabacum*; Bai et al., 2011); and *MdbHLH3* and *MdMYC2* in apple (*Malus domestica*; Espley et al., 2007; An et al., 2016). bHLH TFs interact with MYB and WD40 TFs to form a ternary complex (MBW) that regulates the expression of flavonoid biosynthesis and structural genes (Zhou et al., 2015; Lloyd et al., 2017). VcbHLHL1 was found to interact with VcMYBL1 and VcWDL2 to enhance anthocyanin accumulation and color development in blueberry fruit (*Vaccinium* spp.; Zhao et al., 2019).

Recently, a high-quality genome sequence of *J. regia* was obtained by second- and third-generation sequencing combined with high-throughput chromosome conformation capture and genetic and physical mapping (Zhang et al., 2020); and the Portal of Juglandaceae was established by integrating genome, coding, and amino acid sequences as well as various types of annotation, expression, and microRNA data obtained using BLAST, jbrowse, and other query tools (Guo et al., 2020). Although these resources provide a theoretical basis for genetic improvement of walnut, comparative genomic data of Juglandaceae are still lacking. The present work was carried out in order to identify bHLH TFs in *J. regia* from genome data and characterize their spatiotemporal expression patterns. Our findings provide insight into the roles of bHLHs in the regulation of anthocyanin biosynthesis in red walnut.

## Materials and Methods

### Plant Material

Tissue specimens of red walnut (*J. regia* L. “RW-1”) and common green walnut (*J. regia* L. “Zhonglin 1”) were collected from the Fruit Tree Experimental Station of College of Horticulture, Henan Agricultural University, Zhengzhou, China. The samples were obtained at the following time points: when leaf color changed from red to green; the leaf expansion (NY-1 and RY-1), new shoot growth (NY-2 and RY-2), and fruit swelling (NY-3 and RY-3) stages; and early period of fruit ripening (NY-4 and RY-4). Fruit peels were collected 30 days (NP-1 and RP-1), 60 days (NP-2 and RP-2), and 90 days (NP-3 and RP-3) after flowering. Samples were obtained as three biological replicates from three comparable plants and immediately frozen in liquid nitrogen for transcriptome sequencing as previously described (Li et al., 2018a). A total of 42 libraries were constructed by RNA sequencing (RNA-seq).

In order to maintain a consistent genetic background, different phenotypes (red vs. green leaves) of “RW-1” natural hybrid progeny were selected and cultivated at the Fruit Tree Experimental Station. Because of the long juvenile period of walnut, the progeny have not yet borne fruit; therefore, leaves of different colors were used as the material for experiments. The leaves were sampled at the full red period [new shoot growth stage (SG-1 and SR-1)], red-green period [fruit swelling stage (SG-2 and SR-2)], and full green period [early period of fruit ripening (SG-3 and SR-3); Supplementary Figure S3]. Leaves were sampled as three biological replicates from three comparable plants and wrapped in aluminum foil, placed in liquid nitrogen, and stored at −80°C until use.

*Nicotiana benthamiana* plants used for protein subcellular localization analysis were maintained in growth chambers at 22°C on a 16-h photoperiod.

### RNA-Seq and Library Construction

Total RNA was extracted from leaves and the purity and integrity were analyzed as previously described (Li et al., 2018a). Each 0.5 g sample was prepared as three biological replicates. RNA concentrations were determined using a NanoDrop 1,000 spectrophotometer (Thermo Fisher Scientific, Waltham, MA, United States) and the quality was verified on a 1% agarose gel. Library construction and RNA-seq were performed by Biomarker Biotechnology Corp. (Beijing, China) on a HiSeq 2,500 platform (Illumina, San Diego, CA, United States).

RNA-seq data from different tissues (peel and leaf) of red walnut and common green walnut at different developmental stages were analyzed as described in our previous work (National Center for Biotechnology Information [NCBI] accession no. GSE162007). The heatmap of walnut *bHLH* gene expression patterns was constructed with TBtools (Chen et al., 2020), with the color scale representing fragments per kilobase of transcript per million mapped read counts.

### Identification of *JrbHLH* Genes by Bioinformatic Analysis

Walnut genome annotation information, genome sequences, and protein sequences were obtained from the walnut genome database (Guo et al., 2020).^1^ The coding sequences of all walnut *bHLH* genes were extracted from the genome using TBtools (Liu et al., 2020). A hidden Markov model of the bHLH domain (PF00010) was downloaded from Pfam and used for protein screening with HMMER software (e-value < 0.01; Finn et al., 2011).^2^ bHLH protein sequences of *Arabidopsis* were obtained from The Arabidopsis Information Resource,^3^ and were searched in BLASTP against the common walnut genome database. Candidate proteins obtained by the above methods were merged and confirmed using NCBI Conserved Domains Database (CDD; Marchler-Bauer et al., 2017) and Simple Modular Architecture Research Tool (SMART; Letunic and Bork, 2017).^4^

Identified walnut *bHLH* family genes were analyzed in terms of chromosomal position and gene collinearity based on a published genomic sequence annotation file (Zhang et al., 2020) and mapped using TBtools,^5^ which were also used to determine the nonsynonymous/synonymous mutation (Ka/Ks) ratio of the genes. Conserved *cis*-regulatory elements in the promoter region of walnut *bHLH* genes were identified by analyzing the 2000-bp sequence upstream of the transcription start site obtained from Phytozome online webserver.^6^ Promoter sequence analysis was carried out using PlantCARE.^7^

### Analysis of *JrbHLH* Gene Structure

Information on bHLH sequences was obtained using NCBI BLAST. The theoretical isoelectric point (pI) and molecular weight (MW) were predicted using ExPASy (Artimo et al., 2012).^8^ Cell-PLoc software was used to predict the subcellular localization of the proteins (Chou and Shen, 2008).^9^

The structure of walnut *bHLH* genes was analyzed using TBtools. Conserved motifs in full-length bHLH proteins were identified using the Multiple Expectation Maximization for Motif Elicitation (MEME) program (Bailey et al., 2009) with an optimum motif width ranging from 6 to 200 amino acid residues and a maximum of 10 misfits.^10^ Multiple sequence alignment was performed for the amino acid sequences of walnut bHLH proteins using DNAMAN (Zang et al., 2015).

### Functional Analysis of *JrbHLH* Genes

Blast2GO software (Conesa and Götz, 2008) was used for Gene Ontology (GO) analysis of walnut *bHLH* genes using full-length amino acid sequences. Putative JrbHLH protein sequences were submitted to the online server STRING v10, with *A. thaliana* specified as the organism. The set of genes with the highest bit scores were used to construct the network. The annotation information of the functional domains was manually copied from the BLAST results.

### Phylogenetic Analysis of JrbHLH Protein Sequences

Phylogenetic analysis of bHLH protein sequences of walnut was performed using MEGAX software; bHLH protein sequences are known to be related to anthocyanin biosynthesis in other plant species. A phylogenetic tree was constructed with the maximum likelihood method, with a partial deletion of 1,000 bootstraps and a JTT+F+G model.

### *JrbHLH* Gene Expression Profiling by Quantitative Real-Time (qRT)-PCR

First-stand cDNA was synthesized using the FastQuant RT Kit (with gDNase; Tiangen Biotech, Beijing, China) and stored at −20°C. Primers were designed using Primer Premier 5.0 (Supplementary Table S1). qRT-PCR was performed on an ABI 7500 Real-Time PCR system (Applied Biosystems, Foster City, CA, United States) with the ChamQ Universal SYBR qPCR Master Mix (Vazyme, Nanjing, China). The cDNA was diluted to 200 ng, and 20 μl reactions were prepared as three technical replicates with a 1-μl template per reaction. The qRT-PCR conditions were as follows: 95°C for 5 min, followed by 40 cycles of 95°C for 15 s and 60°C for 1 min, and 72°C for 5 min. The β-actin gene (LOC108996275) was used as an internal reference (Li et al., 2018a). Target gene levels were calculated with the 2^−∆∆Ct^ method (Livak and Schmittgen, 2001).

### *In vitro* Analysis of JrbHLH Protein Subcellular Localization

We generated recombinant JrbHLH proteins for subcellular localization analysis. We first PCR-amplified the coding sequences of the four candidate JrbHLHs related to anthocyanin biosynthesis using specific primers (Supplementary Table S2). Two restriction endonucleases (*Eco*RI and *Kpn*I) were used to digest the amplification products, which were subcloned into the pCAMBIA Super 1,300-green fluorescent protein (GFP) vector using the ClonExpress Ultra One Step Cloning Kit (Vazyme Biotech, Nanjing, China). Positive clones were confirmed by sequencing and transformed into *Agrobacterium tumefaciens* strain GV3101. Cells transformed with the empty vector served as a negative control. Transformed *A. tumefaciens* were infiltrated into the fully expanded leaves of *N. benthamiana*. After 72 h, GFP expression was visualized with an LSM 710 laser confocal microscope (Carl Zeiss, Jena, Germany).

## Results

### Identification and Characterization of Walnut *bHLH* Genes

Using the *Arabidopsis* bHLH protein domain as the query sequence, 192 putative walnut JrbHLH protein sequences were obtained with default parameters using HMMER and BLASTP. After removing redundant sequences, 102 *bHLH* genes with a conserved bHLH domain were identified in walnut and confirmed in NCBI CDD and with SMART (Supplementary Table S3).

The physiochemical properties of the 102 *JrbHLH* genes, including amino acids, length of coding sequences, theoretical pI, predicted MW, and predicted subcellular localization, are shown in Supplementary Table S3. Sequence analysis revealed that the *JrbHLH* genes encoded proteins ranging in length from 156 (JrLAX) to 842 (JrbHLH157) amino acids, with theoretical pI values ranging from 4.78 (JrUNE12c) to 10.46 (JrbHLH131). JrbHLH36a and JrbHLH131 were predicted to localize to mitochondria, while all other JrbHLHs were predicted to localize to the nucleus.

### Gene Structure, Conserved Motifs, and Multiple Sequence Alignment

To elucidate the structure and functions of *JrbHLH* genes, we analyzed their conserved motifs and gene structure (Supplementary Figure S4). The most highly conserved *JrbHLH* genes shared a common structure, with the number of introns ranging from 0 (17 genes were without introns) to 9 (*JrbHLH155* and *JrbHLH157*; Supplementary Figure S4C). In the phylogenetic analysis, the 102 bHLHs were divided into 15 groups according to conserved domains, and 10 conserved protein motifs were identified using MEME (Supplementary Figures S4A,B). Among them, all sequences were found to exhibit two types of highly conserved protein motifs which are demonstrated as green (motif 1) and yellow (motif 2) blocks, respectively; and these two conserved domains were adjacent to each other. Motif 1 of JrbHLH was composed of basic residues and loop helix 1, while motif 2 comprised a loop and helix 2 (Supplementary Figures S4B, S5). The gap between motifs 1 and 2 varied depending on loop length. The sequences of the basic region and the position of the two helix domains were more conserved compared with the sequences obtained from the loop region, and the conserved motif model of the bHLH proteins in walnut were E-R-R-R-L-L-P-L-L.

### Chromosomal Location and Gene Collinearity Analysis of Walnut bHLH Family

**Figure 1:**
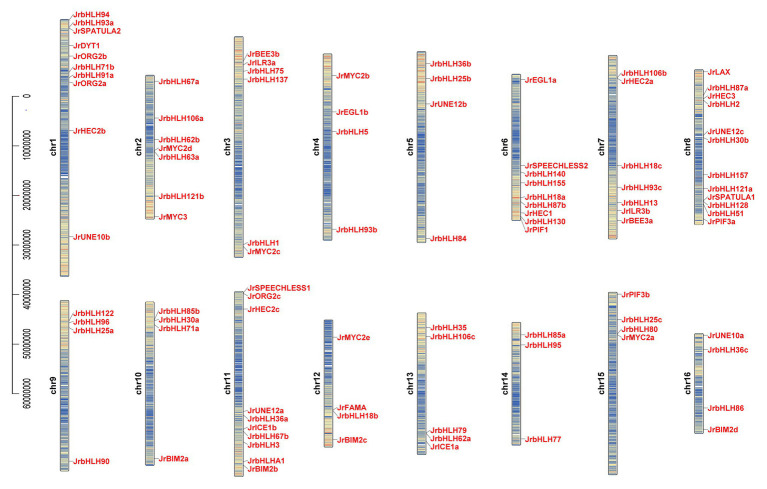
Gene location and distribution of walnut *bHLH* genes on chromosomes. There are 16 chromosomes (Chr1-Chr16) in the walnut genome (2n = 32). Gene positions and chromosome length were measured using the scale on the left in mega bases (bp). Red indicates high density and blue indicates low density.

We analyzed the chromosomal location and distribution of *JrbHLH* genes in the walnut genome (Figure 1). The density of gene distribution on each chromosome is basically higher on both sides, but lower in the middle. Chromosome 8 harbored the largest number of *JrbHLH* genes (12), followed by chromosomes 1 and 11 (10 each); chromosomes 4, 5, 9, 10, 12, 15, and 16 (4 each); and chromosome 14 (3). To investigate whether segmental duplication contributed to the expansion of the walnut *bHLH* gene family during evolution, we mapped the genes onto duplicated walnut chromosome blocks (Supplementary Figure S6) and found that 42 *JrbHLH* genes comprising 21 paralogous pairs and accounting for 41.2% of the entire gene family were located on the duplicated blocks. Genes were replicated on all 16 chromosomes; chromosomes 7, 8, 9, and 10 had the most genes (4 each), followed by chromosomes 5, 6, 13, and 14 (3 each). Gene clustering was observed on chromosomes 8 (*JrbHLH87a* and *JrbHLH2*), 9 (*JrbHLH122*, *JrbHLH96*, and *JrbHLH25a*), and 10 (*JrbHLH85b*, *JrbHLH30a*, and *JrbHLH71a*). These results suggest that the expansion of the walnut bHLH gene family occurred through tandem duplication. The Ka/Ks ratio can serve as an indicator of selection pressure on a gene during evolution; the ratio was <1 for all duplicated *bHLH* gene pairs in red walnut (Table 1), indicating that the genes primarily evolved under the influence of purifying selection.

**Table 1:** Estimated Ka/Ks ratios of the duplicated *bHLH* genes in walnut.

No.	Paralogous pairs	Ka^a^	Ks^b^	Ka/Ks	Effective length (bp)	Average S-sites^c^	Average N-sites^d^
1	JrbHLH106b/JrbHLH51	0.326360603	1.142289974	0.285707317	1,032	232.75	799.25
2	JrUNE10a/JrPIF3b	0.049684901	0.321881900	0.154357548	1,032	231.17	800.83
3	JrbHLH71a/JrbHLH25a	0.243548876	0.592141481	0.411301832	609	135.75	473.25
4	JrbHLH95/JrbHLH106c	0.093581793	0.284617216	0.328798777	1,629	367.33	1,261.67
5	JrbHLH87b/JrbHLH25b	0.090287139	0.291450977	0.309784992	1,305	294.00	1,011.00
6	JrbHLH18b/JrbHLH36a	0.074034048	0.325261057	0.227614240	900	209.67	690.33
7	JrbHLH87a/JrILR3b	0.199232627	0.293311064	0.679253707	1,086	235.83	850.17
8	JrbHLH121b/JrUNE10b	0.129739996	0.314042048	0.413129379	1,209	268.92	940.08
9	JrbHLH13/JrbHLH2	0.115204849	0.268147893	0.429631752	975	218.08	756.92
10	JrbHLH30a/JrbHLH96	0.055664234	0.316470246	0.175890892	702	163.00	539.00
11	JrICE1a/JrbHLH77	0.110981775	0.379320251	0.292580674	1,179	258.92	920.08
12	JrBIM2a/JrbHLH90	0.077668165	0.562361025	0.138110860	459	99.83	359.17
13	JrbHLH18c/JrbHLH30b	0.137014698	0.573216014	0.239028036	2,088	488.25	1,599.75
14	JrbHLH75/JrMYC2b	0.109689265	0.409838281	0.267640360	984	218.00	766.00
15	JrUNE12b/JrbHLH155	0.101273304	0.300035299	0.337537964	993	222.42	770.58
16	JrSPATULA2/JrbHLH67a	0.166577149	0.442884592	0.376118637	765	171.42	593.58
17	JrbHLH36c/JrMYC2a	0.152510115	0.887189546	0.171902515	711	156.67	554.33
18	JrBIM2b/JrBIM2c	0.064199573	0.388788791	0.165127119	675	154.92	520.08
19	JrbHLH122/JrbHLH85b	0.203625451	0.375423255	0.542389021	687	158.00	529.00
20	JrbHLH84/JrEGL1a	0.111058610	0.385308396	0.288233038	1,002	235.08	766.92
21	JrbHLH35/JrbHLH85a	0.130131537	0.369838976	0.351859987	1,365	306.83	1,058.17

a Non-synonymous substitution rate.

b Synonymous substitution rate.

c The average number of synonymous sites.

d The average number of non-synonymous sites.

### Pivotal *cis*-Elements in the Promoters of Walnut *bHLH* Genes

We investigated gene regulation patterns by analyzing *cis*-regulatory elements in the 2000 bp upstream of the transcription start site (promoter region) of *JrbHLH* genes using PlantCARE (Supplementary Figure S7 and Supplementary Table S4). There were 16 functionally annotated *cis*-regulatory elements in the promoters of most *JrbHLHs* that were roughly divided into three categories: light-responsive elements (Box 4, G-box, GT1 motif, TCT motif, and I-box); stress-responsive elements (antioxidant response element, MYB-binding site [MBS], and long terminal repeat); and hormone-responsive elements (abscisic acid-responsive element, CGTCA motif, TGACG motif, O2-site, TCA element, TGA element, and P-box). The presence of MBSs in the *JrbHLH* gene promoter suggests that bHLH proteins in red walnut interact with MYB TFs to modulate the expression of downstream targets.

### Gene Ontology Annotations

To analyze the functions of walnut bHLH TFs, *Arabidopsis* was used as model species for GO analysis with Blast2Go (Supplementary Figure S8). In the biological process category, *JrbHLH* genes were significantly enriched in biological regulation (*n* = 16, 29%), cellular process (*n* = 14, 25%), metabolic process (*n* = 10, 18%), single-organism process (*n* = 6, 11%), developmental process (*n* = 4, 7%), response to stimulus (*n* = 2, 4%), multicellular organismal process (*n* = 2, 4%), and signaling (*n* = 1, 2%). In the cellular component category, the genes were enriched in cell (*n* = 15, 24%), cell part (*n* = 15, 24%), organelle (*n* = 15, 24%), organelle part (*n* = 8, 13%), macromolecular complex (*n* = 8, 13%), and membrane (1, 2%). In the molecular function category, the genes were enriched in binding (*n* = 69, 80%), nucleic acid-binding TF activity (*n* = 13, 15%), and catalytic activity (*n* = 4, 5%). Thus, the functions of *JrbHLH* genes mainly involve nucleic acid-binding TF activity and catalytic activity in biological regulation; cellular, single-organism, and developmental processes; and metabolism. Some JrbHLH proteins were identified as components of macromolecular complexes.

### Protein Interaction Network

**Figure 2:**
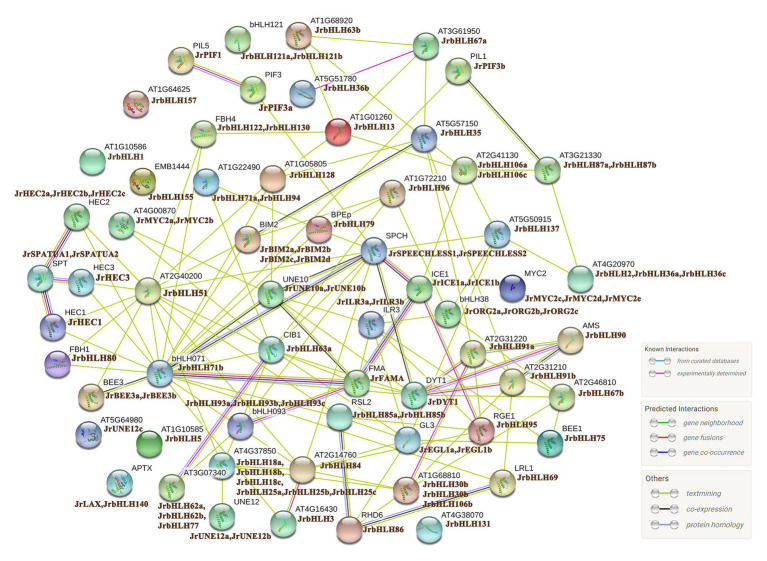
Protein interaction network for JrbHLHs according to JrbHLH orthologs in Arabidopsis. The online tool STRING was used to predict the network. JrbHLH proteins are shown on the side with Arabidopsis orthologs.

bHLH family members generally function by forming homo- or heterodimers with other proteins, which is critical for their binding to target gene promoters (Carretero-Paulet et al., 2010). We analyzed orthologous AtbHLH proteins to construct a PPI network of the 102 candidate JrbHLH proteins (Figure 2). The interactions between proteins were established from known interactions (from curated databases and experimental findings) and predictions (ie, based on neighboring genes, gene fusions, and gene co-occurrence) using various tools (text mining and co-expression and protein homology analyses). Thirteen of the bHLH TFs, JrbHLH121a, JrbHLH121b, JrbHLH57, JrbHLH1, JrbHLH155, JrUNE12c, JrbHLH5, JrLAX, JrbHLH140, JrbHLH131, JrMYC2c, JrMYC2d, and JrMYC2e, did not interact with other family members. On the other hand, over 30 proteins interacted with at least 4 other bHLH proteins, with JrbHLH71b having the largest number of interaction partners. These 30 JrbHLH proteins – especially JrbHLH71b – are presumed to play important roles in plant growth and development.

### Screening of the Candidate *bHLH* Genes by Phylogenetic Analysis

**Figure 3:**
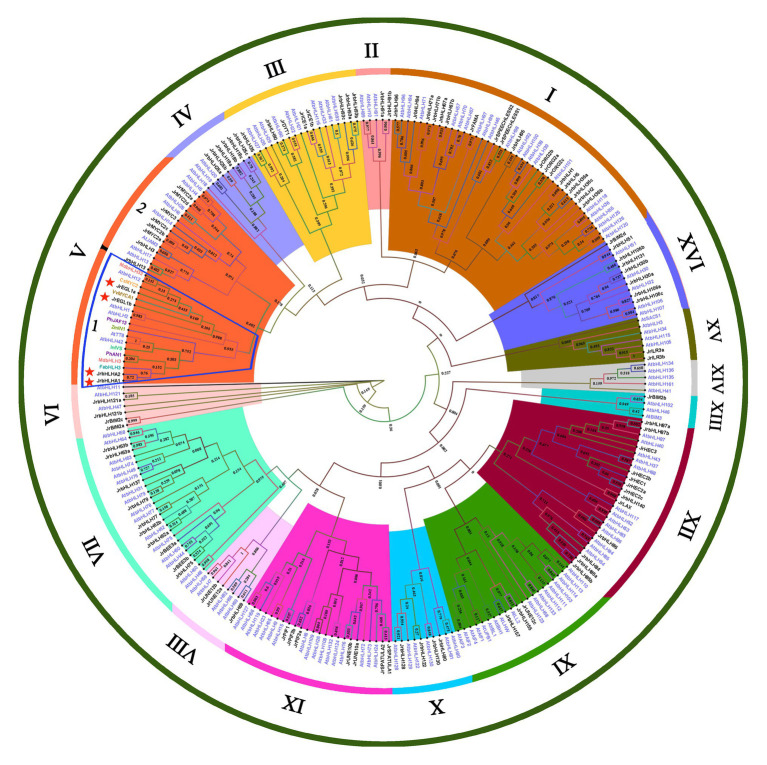
Phylogenetic tree analysis of bHLH proteins between different species. Phylogenetic tree constructed with bHLHs of walnut, *Arabidopsis thaliana*, and proteins related to anthocyanin biosynthesis in other species, including *Fragaria* × *ananassa* FabHLH3 (AFL02463.1), *Malus domestica* MdbHLH3 (ADL36597.1) and MdbHLH33 (ABB84474.1), *Petunia* × *hybrida* PhAN1 (AAG25927.1), and PhJAF13 (AAC39455.1), *Ipomoea nil* InIVS (BAE94394.1), *Zea mays* ZmIN1 (AAB03841.1), *Citrus sinensis* CsMYC2 (ABR68793.1), and *Vitis vinifera* VvMYCA1 (NP_001267954.1).

To investigate the evolutionary relationships and functions of red walnut bHLH proteins and identify those that are involved in anthocyanin biosynthesis, we constructed a phylogenetic tree comprising the 102 JrbHLH proteins along with 145 AtbHLH proteins and 10 proteins related to this process in other plant species (Figure 3). The proteins were classified into 16 subfamilies (I-XVI) based on the classification in *Arabidopsis* (Toledo-Ortiz et al., 2003). Group I was largest with 40 proteins, while group XIII was the smallest with 4 proteins. There were no walnut bHLH proteins in group XIV. bHLH proteins related to anthocyanin biosynthesis were concentrated in group V-1; 4 from *J. regia* were in this group (JrEGL1a, JrEGL1b, JrbHLHA1, and JrbHLHA2). JrEGL1a and JrEGL1b showed the highest degree of homology with VvMYCA1 and cucumber (*Cucumis sativus*) MYC2, while JrbHLHA1 and JrbHLHA2 were most closely related to strawberry (*Fragaria* × *ananassa*) *bHLH3*. Thus, these 4 *JrbHLH* genes are potentially involved in anthocyanin biosynthesis in red walnut.

### Expression Pattern of the Walnut *bHLH* Genes in Different Tissues of Red Walnut and Common Green Walnut

**Figure 4:**
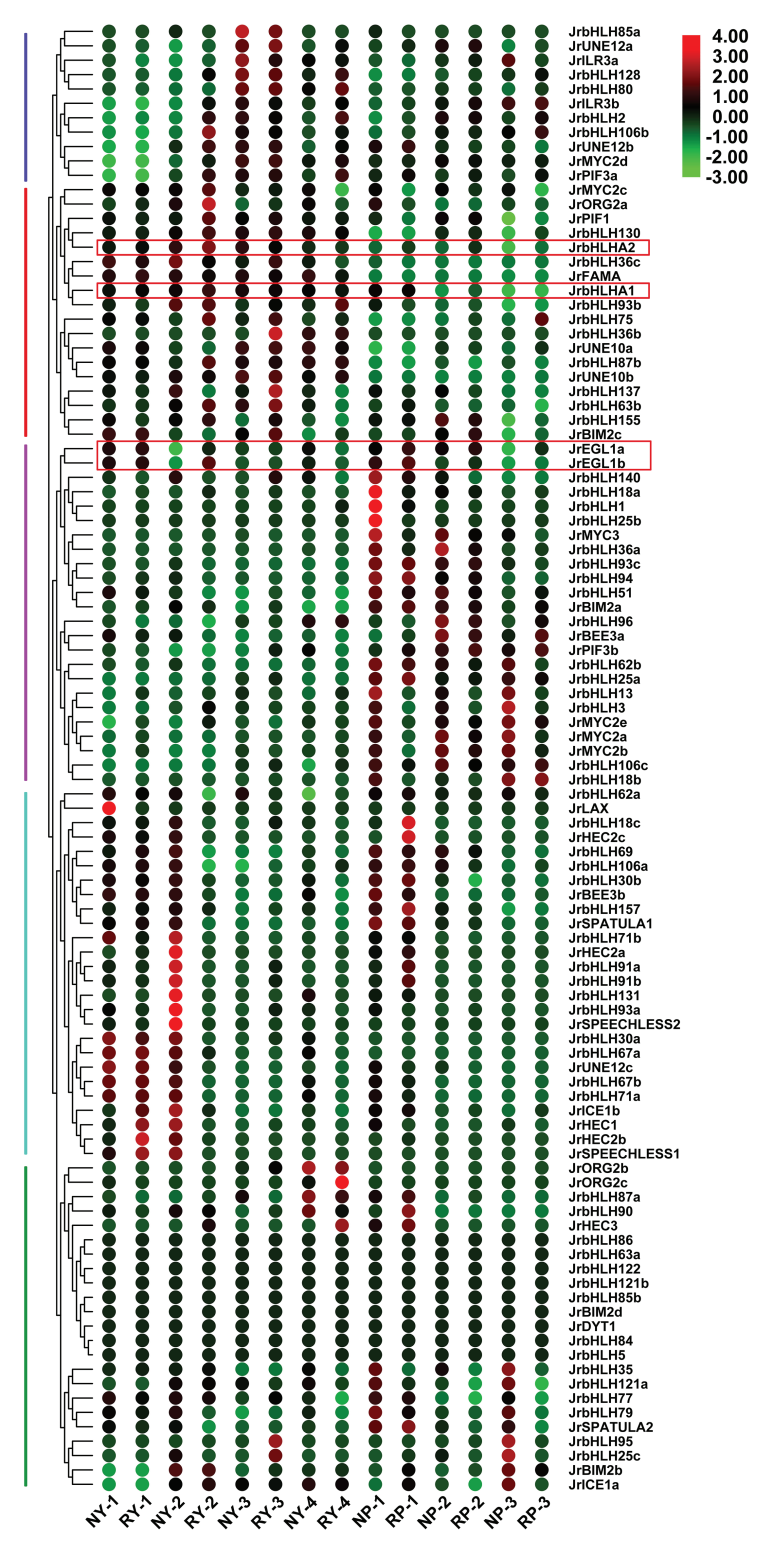
Gene expression pattern of *JrbHLHs* in different tissues (leaves and peels) of red walnut and common green walnut by RNA-seq. Leaves of the red walnut and common green walnut were collected at four stages. NY, common green walnut leaves; RY, red walnut leaves. (1) Leaf-expansion stage; (2) new shoot growing stage; (3) fruit swelling stage; and (4) early period of fruit ripening. Peels of the red walnut and common green walnut were collected at three stages. NP, common green walnut peel; RP, red walnut peel. 1–3, 30, 60, and 90 days after flowering, respectively. The scale bars represent the log2 transformations of the RPKM values.

The expression patterns of *bHLH* genes at different developmental stages were analyzed in different tissues (leaf and peel) of red walnut and common green walnut (Figure 4) using published RNA-seq data (NCBI accession no. GSE162007; Li et al., 2018a). The genes were divided into 5 groups based on expression pattern. *JrbHLH* genes in group 1 were more highly expressed in the late stages of leaf and peel development than at the early stages, whereas the opposite trend was observed in group 4. Genes in group 2 showed elevated expression at different stages of leaf development but had lower expression in peel; conversely, genes in group 3 showed low expression in leaves but were highly expressed in the peel. Nine genes in group 5 (*JrbHLH86*, *JrbHLH63a*, *JrbHLH122*, *JrbHLH121b*, *JrbHLH85b*, *JrBIM2d*, *JrDYT1*, *JrbHLH84*, and *JrbHLH5*) showed no differences in expression between tissues in red walnut and common green walnut. Genes related to anthocyanin biosynthesis (*JrEGL1a*, *JrEGL1b*, *JrbHLHA1*, and *JrbHLHA2*) showed similar expression patterns in the leaf and peel of red walnut and common green walnut at different developmental stages.

### Expression Pattern of the Walnut *bHLH* Genes in Different Phenotypic Leaves of Red Walnut Natural Hybrid Progeny

We compared the functions of *JrbHLH* genes during the development of different phenotypes of red walnut natural hybrid progeny (red vs. green leaves) by analyzing the associated gene expression profiles obtained by RNA-seq. A total of 43,291,604–62,191,784 raw reads and 22,193,238–31,095,892 clean reads were obtained from the 18 libraries. The percentage of bases with a quality score of Q30 was ≥95.22%, indicating that the sequencing results were reliable and could be used for subsequent analysis (Supplementary Table S5).

**Figure 5:**
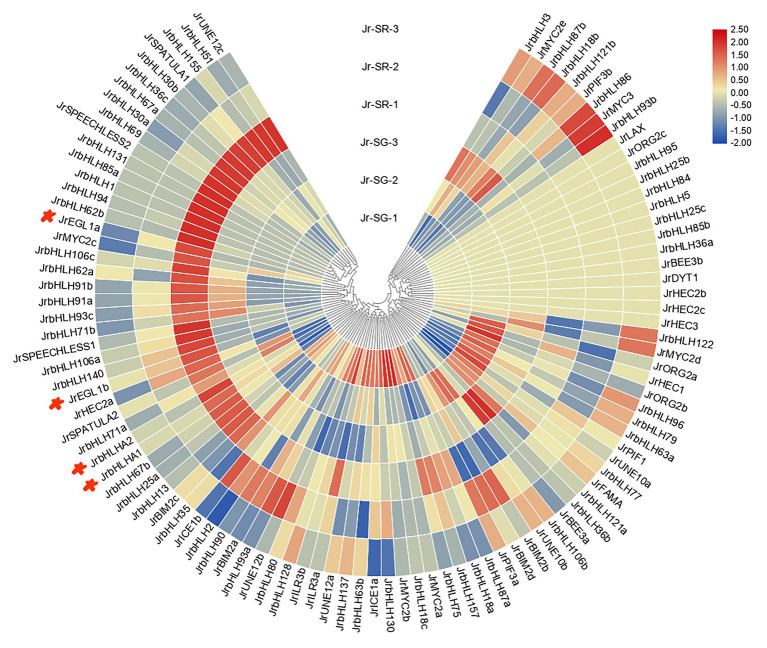
Gene expression pattern of *JrbHLHs* in different phenotypic (red leaves and green leaves) of red walnut natural hybrid progeny by RNA-seq. Leaves of the different phenotypes (red leaves and green leaves) of red walnut natural hybrid progeny were collected at three stages. SG, Seedling progenies-Green leaves; SR, Seedling progenies-Red leaves. (1) The full red period (vigorous growth period of new shoots); (2) the red-green period (seed filling period); and (3) the whole green period (early period of fruit ripening). The scale bars represent the log2 transformations of the RPKM values.

The *JrbHLH* genes had distinct expression patterns in different colored leaves of red walnut progeny (Figure 5). The genes were divided into 4 groups according to expression level; expression patterns within each group were similar. The differential expression indicated functional divergence of *JrbHLH* genes although some (*JrLAX*, *JrORG2c*, *JrbHLH95*, *JrbHLH25b*, *JrbHLH84*, *JrbHLH5*, *JrbHLH25c*, *JrbHLH85b*, *JrbHLH36a*, *JrBEE3b*, *JrDYT1*, *JrHEC2b*, *JrHEC2c*, and *JrHCE3*) were expressed at comparable levels in all samples. There were 37 *JrbHLH* genes – including the candidate anthocyanin biosynthesis-related genes *JrEGL1a*, *JrEGL1b*, *JrbHLHA1*, and *JrbHLHA2* – that showed maximum expression in the darkest period of red leaves (SR-1, the full red period of red leaves in seedling progeny) and lower expression during other periods, suggesting that these genes are responsible for the red color of walnut leaves.

### Gene Expression Analysis by qRT-PCR

**Figure 6:**
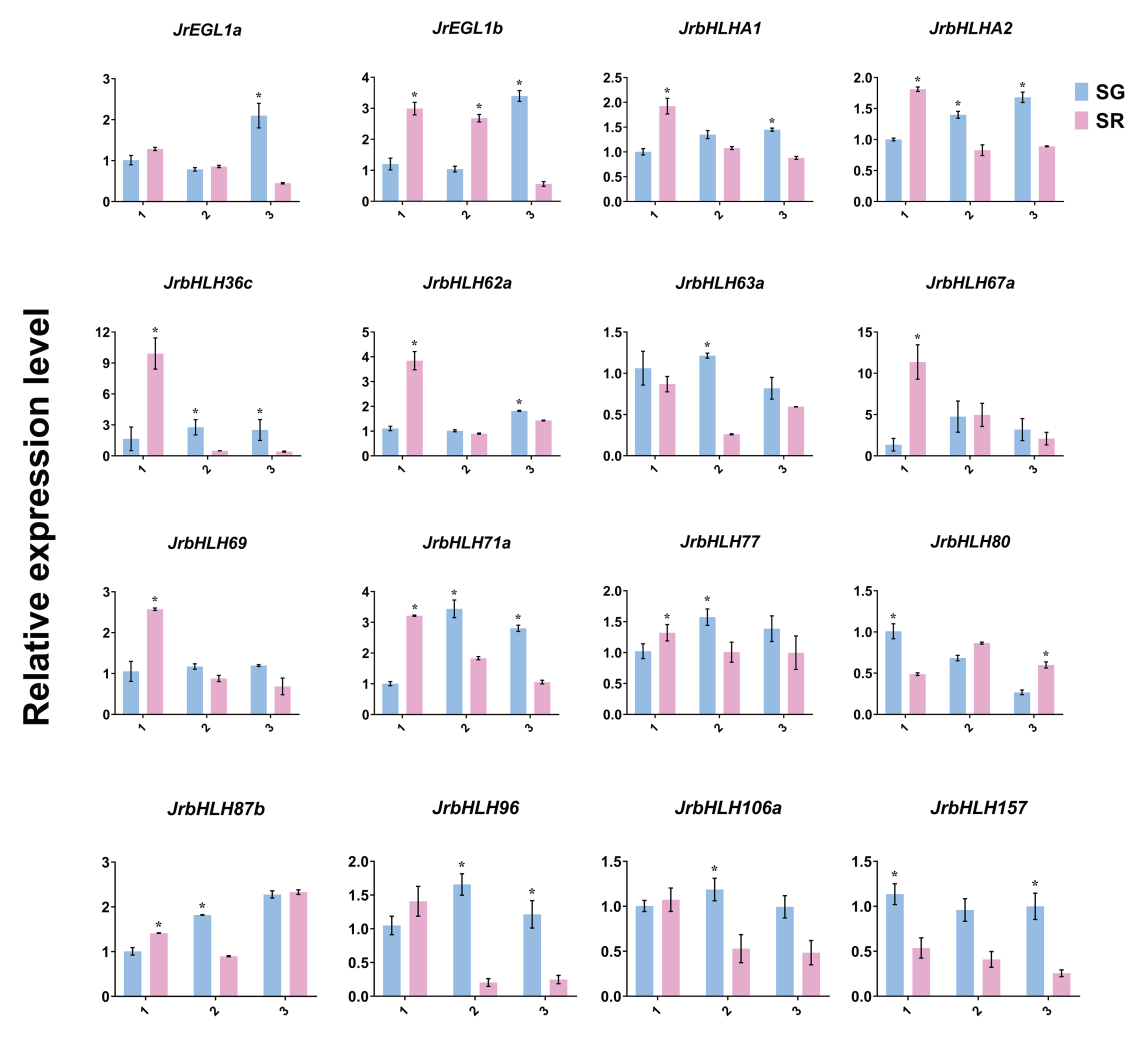
Expression of the 16 *JrbHLHs* in the different phenotypes (red leaves and green leaves) of the red walnut natural hybrid progeny. The relative expressions were detected by qRT-PCR. Leaves of the different phenotypes (red leaves and green leaves) of red walnut natural hybrid progeny were collected at three stages. SG, Seedling progenies-Green leaves; SR, Seedling progenies-Red leaves. (1) The full red period (vigorous growth period of new shoots); (2) the red-green period (seed filling period); and (3) the whole green period (early period of fruit ripening). Significant differences were determined using a one-sided paired *t*-test (^*^p < 0.05). Expression values (±SE) of three replicates were normalized using *JrActin* as the internal control.

To further investigate the relationship between *JrbHLH* genes and leaf color in red walnut, the expression levels of 16 candidate genes that were found to be differentially expressed by RNA-seq were analyzed by qRT-PCR (Figure 6). The genes showed variable expression at different developmental stages according to leaf color. At stage 1, 10 genes – namely, *JrEGL1b*, *JrbHLHA1*, *JrbHLHA2*, *JrbHLH36c*, *JrbHLH62a*, *JrbHLH67a*, *JrbHLH69*, *JrbHLH71a*, *JrbHLH77*, and *JrbHLH87b* were more highly expressed in red leaves (SR) than in green leaves (SG), while the opposite was true for *JrbHLH80* and *JrbHLH157*. Meanwhile, *JrEGL1a*, *JrbHLH63a*, *JrbHLH96*, and *JrbHLH106a* showed no differences in expression levels between SR and SG at this stage. With the exception of *JrEGL1b*, which continued to be expressed at a higher level in SR than in SG, at stage 2 the expression of *JrbHLH* genes were higher in green leaves, with significant differences observed for 8 of the genes (*JrbHLHA2*, *JrbHLH36c*, *JrbHLH63a*, *JrbHLH71a*, *JrbHLH77*, *JrbHLH87b*, *JrbHLH96*, and *JrbHLH106*). At stage 3, *JrbHLH* genes were more highly expressed in SG than in SR except for *JrbHLH80* and *JrbHLH87b*, with 9 of the genes (*JrEGL1a*, *JrEGL1b*, *JrbHLHA1*, *JrbHLHA2*, *JrbHLH36c*, *JrbHLH62a*, *JrbHLH71a*, *JrbHLH96*, and *JrbHLH157*) showing significant differences. The expression levels of the remaining *bHLH* genes did not differ between SR and SG at this stage. Pearson correlation analysis showed a strong correlation (*R*^2^ = 0.8292) between RNA-seq data and qRT-PCR results (Supplementary Figure S9), supporting the validity of our data.

### Subcellular Localization of JrbHLH Proteins

**Figure 7:**
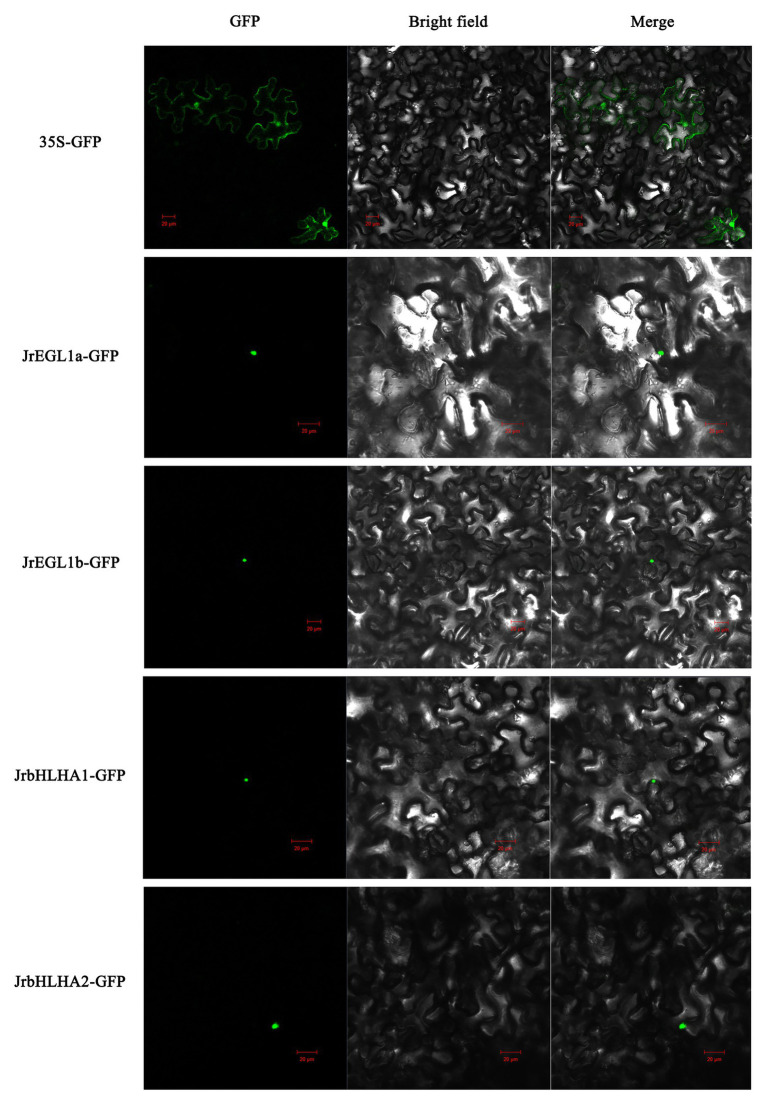
Subcellular localization of JrbHLH proteins. Transient expression of Super1300 construct and Super1300-JrbHLH fusion in tobacco epidermal cells by *Agrobacterium*-mediated transient transformation; the GFP signal was observed by confocal laser microscopy. From left to right, the pictures show GFP, bright-field, and merged. Bar = 20 μm.

We examined the subcellular localization of the four candidate anthocyanin biosynthesis-related TFs (JrEGL1a, JrEGL1b, JrbHLHA1, and JrbHLHA2) in *N. benthamiana* transiently infiltrated with plasmids encoding the cDNA of the corresponding genes. The GFP reporter protein was detected in both the nucleus and cytoplasm in the leaves of control plants; however, in plants transfected with the four JrbHLH-GFP fusion constructs, there was nuclear accumulation of the GFP signal (Figure 7).

## Discussion

Anthocyanins are plant secondary metabolites that play essential roles in plant growth and development and stress tolerance (Meng et al., 2020). The biosynthesis of anthocyanin depends on phenylalanine metabolism (Supplementary Figure S2) and involves the regulation of structural genes by the MBW complex formed by MYB, bHLH, and WD40 TFs (Baudry et al., 2004; Ma et al., 2018). In apple, the upregulation of MdMYB1 mediated by MdbHLH3 amplified the regulatory signal for anthocyanin biosynthesis (Xie et al., 2012), while in mulberry, abnormal expression of bHLH3 disrupted flavonoid homeostasis, resulting in variable pigmentation (Li et al., 2020). However, the bHLH TFs involved in anthocyanin biosynthesis have not been reported in walnut. In this study, we identified 102 *bHLH* genes in the walnut genome, and their predominantly nuclear localization was in accordance with their function as TFs (Supplementary Table S3). *bHLH* genes constitute large families, with 188 members in apple and 138 in jujube (*Ziziphus jujuba*; Mao et al., 2017; Shi et al., 2019) that can be divided into 15–25 subfamilies (Pires and Dolan, 2010). Our phylogenetic analysis showed that walnut bHLHs formed 15 subfamilies (Supplementary Figure S4A and Figure 3; Heim et al., 2003). Interestingly, there were no JrbHLH proteins in group XIV based on the *Arabidopsis* classification; similar results have been reported for safflower (*Carthamus tinctorius*), which lacks members in subfamilies 5, 8, 15, 18, and 21 that may have been lost over the course of evolution (Hong et al., 2019). One of the conserved motifs of bHLH proteins in walnut contains several highly conserved residues – namely, E (Glu), R (Arg), and L (Leu) – that are required for binding to target DNA sequences (Supplementary Figure S4B; Toledo-Ortiz et al., 2003). By sequence alignment we identified 2 main conserved domains in the bHLH protein, the basic region and helix region with loop; additionally, a conserved proline marks the beginning of the first helix (Supplementary Figure S5). Our results are consistent with previous reports (Toledo-Ortiz et al., 2003; Hong et al., 2019).

The expansion of gene families during evolution is mainly the result of duplication events. *JrbHLH* genes were present on each chromosome but were unevenly distributed (Figure 1). *In silico* mapping of the genes to chromosomes suggested a large number of gene duplication events including segmental and tandem duplication, which likely played an important role in the expansion of the walnut *bHLH* gene family (Supplementary Figure S6 and Table 1).

The promoter of a gene contains *cis*-regulatory elements that can potentially reveal gene function (Xie et al., 2020). Walnut *bHLH* genes were found to contain three types of *cis*-acting elements, light-, stress-, and hormone-responsive elements (Supplementary Figure S7). Moreover, *JrbHLH* gene promoters contained the bHLH binding site (G-box) and MBS, indicating that JrbHLH TFs can interact with each other or with MYB TFs in the regulation of walnut growth and development. This was confirmed by the enrichment in binding activity (Supplementary Figure S8) in GO annotations as well as the PPI network (Figure 2).

Most *JrbHLHs* showed distinct spatial and temporal expression patterns (Figures 4, 5), suggesting varied functions in walnut growth and development. *JrEGL1a*, *JrEGL1b*, *JrbHLHA1*, and *JrbHLHA2* belonging to group V-1 clustered together in a subgroup containing anthocyanin-related *bHLH* genes of several plant species (Figure 3; Cultrone et al., 2010; Matus et al., 2010; Chen et al., 2016). RNA-seq confirmed that these four genes were highly expressed during the color change stages of leaf and peel development in red walnut, and in red leaves of red walnut natural hybrid progeny, but had lower expression in common green walnut and green leaves of red walnut progeny at the stage where the phenotypic difference was greatest (Figures 4, 5). This was supported by qRT-PCR analysis of genes that were differentially expressed in red walnut natural hybrid progeny with different leaf colors (Figure 6). As leaf development progressed, the difference in leaf color increased whereas the differential gene expression decreased, such that *JrbHLHA2* was more highly expressed in green as compared to red leaves. We speculate that at this point the biosynthesis of anthocyanin was blocked and its degradation accelerated in red leaves, resulting in the downregulation of the *JrbHLH* gene, reduced anthocyanin accumulation, and a color change from red to green. At the late stage of leaf development, anthocyanin biosynthesis stabilized and the expression of *JrbHLH* genes was upregulated in green leaves. Other TFs besides JrbHLH proteins may participate in the regulation of anthocyanin content in walnut. However, the difference in peel color between red walnut and common green walnut increased over the course of development, with a correspondingly higher expression of *JrbHLH* genes in the former (Figure 4). Taken together, our results indicate that JrEGL1a, JrEGL1b, JrbHLHA1, and JrbHLHA2 regulate anthocyanin biosynthesis in walnut, although the detailed mechanisms require further investigation.

## Conclusion

This is the first comprehensive and systematic genome-wide analysis of the *J. regia* *bHLH* gene superfamily. We identified 102 *JrbHLH* genes and characterized their structure and evolutionary conservation. The genes were located on the 16 chromosomes of *J. regia* and could be classified into 15 subfamilies. We determined that 42 of the *JrbHLH* genes were involved in the expansion of the walnut bHLH family, and phylogenetic comparisons with other plant species revealed four *JrbHLH* genes (*JrEGL1a*, *JrEGL1b*, *JrbHLHA1*, and *JrbHLHA2*) that are likely involved in anthocyanin biosynthesis in walnut. This was supported by *in silico* functional enrichment and PPI analyses, as well as RNA-seq and qRT-PCR analyses of genes that were differentially expressed between red walnut and common green walnut and between red vs. green leaves of red walnut natural hybrid progeny at various developmental stages. Our findings provide a basis for future investigations on the molecular mechanisms of anthocyanin biosynthesis in red walnut.

## Data Availability Statement

The datasets presented in this study can be found in online repositories. The name of the repository and accession numbers can be found at: National Center for Biotechnology Information, https://www.ncbi.nlm.nih.gov/, GSE162007 and PRJNA688391.

## Author Contributions

GW designed the research. WZ and YL performed experimental works and data analysis, and prepared the original draft. LL and LW participated in data analysis. HM and YY helped review and editing the draft. ZD provided experimental materials. LW and GW provided support for projects and funds, and revised the manuscript. All authors contributed to the article and approved the submitted version.

### Conflict of Interest

ZD was employed by the Shangluo Shengda Industrial Co., Ltd.

The remaining authors declare that the research was conducted in the absence of any commercial or financial relationships that could be construed as a potential conflict of interest.

## Footnotes

^1^http://www.juglandaceae.net/

^2^http://pfam.xfam.org/

^3^https://www.arabidopsis.org/index.jsp

^4^http://smart.embl-heidelberg.de/

^5^http://xhhuanglab.cn/data/juglans.html

^6^https://phytozome.jgi.doe.gov

^7^http://bioinformatics.psb.ugent.be/webtools/plantcare/html/

^8^http://web.expasy.org/compute_pi

^9^http://www.csbio.sjtu.edu.cn/bioinf/Cell-PLoc/

^10^http://meme-suite.org/meme/

## Abbreviations

bHLH: basic helix-loop-helix
TFs: Transcription factors
PI: Isoelectric points
MW: Molecular weight
Kda: Killi dalton
MW: Molecular weight
CDS: Coding sequences
HMM: Hidden Markov model
GDB: Genome database
CDD: Conserved domain
DEGs: Differentially expressed genes
qRT-PCR: Quantitative real-time PCR

## References

[ref1] An J. P. Li H. H. Song L. Q. Su L. Liu X. You C. X.. (2016). The molecular cloning and functional characterization of MdMYC2, a bHLH transcription factor in apple. Plant Physiol. Biochem. 108, 24–31. 10.1016/j.plaphy.2016.06.032, PMID: 27404131

[ref2] Artimo P. Jonnalagedda M. Arnold K. Baratin D. Csardi G. De Castro E.. (2012). ExPASy: SIB bioinformatics resource portal. Nucleic Acids Res. 40, W597–W603. 10.1093/nar/gks400, PMID: 22661580 PMC3394269

[ref3] Bai Y. H. Pattanaik S. Patra B. Werkman J. R. Xie C. H. Yuan L. (2011). Flavonoid-related basic helix-loop-helix regulators, NtAn1a and NtAn1b, of tobacco have originated from two ancestors and are functionally active. Planta 234, 363–375. 10.1007/s00425-011-1407-y, PMID: 21484270

[ref5] Bailey T. L. Boden M. Buske F. A. Frith M. Grant C. E. Clementi L.. (2009). MEME suite: tools for motif discovery and searching. Nucleic Acids Res. 37, W202–W208. 10.1093/nar/gkp335, PMID: 19458158 PMC2703892

[ref4] Bailey P. C. Martin C. Toledo-ortiz G. Quail P. H. Huq E. Heim M. A. (2003). Update on the basic helix-loop-helix transcription factor gene family in *Arabidopsis thaliana*. Plant Cell 15, 2497–2501. 10.1105/tpc.151140, PMID: 14600211 PMC540267

[ref6] Baudry A. Heim M. A. Dubreucq B. Caboche M. Weisshaar B. Lepiniec L. (2004). TT2, TT8, and TTG1 synergistically specify the expression of *BANYULS* and proanthocyanidin biosynthesis in *Arabidopsis thaliana*. Plant J. 39, 366–380. 10.1111/j.1365-313X.2004.02138.x, PMID: 15255866

[ref7] Butelli E. Licciardello C. Zhang Y. Liu J. J. Mackay S. Bailey P.. (2012). Retrotransposons control fruit-specific, cold-dependent accumulation of anthocyanins in blood oranges. Plant Cell 24, 1242–1255. 10.1105/tpc.111.095232, PMID: 22427337 PMC3336134

[ref8] Carretero-Paulet L. Galstyan A. Roig-Villanova I. Martínez-García J. F. Bilbao-Castro J. R. Robertson D. L. (2010). Genome-wide classification and evolutionary analysis of the bHLH family of transcription factors in Arabidopsis, poplar, rice, moss, and algae. Plant Physiol. 153, 1398–1412. 10.1104/pp.110.153593, PMID: 20472752 PMC2899937

[ref9] Chandler V. L. Radicella J. P. Robbins T. P. Chen J. Turks D. (1989). Two regulatory genes of the maize anthocyanin pathway are homologous: isolation of B utilizing R genomic sequences. Plant Cell 1, 1175–1183. 10.1105/tpc.1.12.1175, PMID: 2535537 PMC159853

[ref10] Chen C. J. Chen H. Zhang Y. Thomas H. R. Frank M. H. He Y. H.. (2020). TBtools - an integrative toolkit developed for interactive analyses of big biological data. Mol. Plant 13, 1194–1202. 10.1016/j.molp.2020.06.009, PMID: 32585190

[ref11] Chen J. X. Mao L. C. Mi H. B. Lu W. J. Ying T. J. Luo Z. S. (2016). Involvement of abscisic acid in postharvest water-deficit stress associated with the accumulation of anthocyanins in strawberry fruit. Postharvest Biol. Technol. 111, 99–105. 10.1016/j.postharvbio.2015.08.003

[ref12] Chou K. Shen H. (2008). Cell-PLoc: a package of web-servers for predicting subcellular localization of proteins in various organisms. Nat. Protoc. 3, 153–162. 10.1038/nprot.2007.494, PMID: 18274516

[ref13] Conesa A. Götz S. (2008). Blast2GO: a comprehensive suite for functional analysis in plant genomics. Int. J. Plant Genom. 2008:619832. 10.1155/2008/619832, PMID: 18483572 PMC2375974

[ref14] Cultrone A. Cotroneo P. S. Reforgiato Recupero G. (2010). Cloning and molecular characterization of R2R3-MYB and bHLH-MYC transcription factors from *Citrus sinensis*. Tree Genet. Genomes 6, 101–112. 10.1007/s11295-009-0232-y

[ref15] Espley R. V. Hellens R. P. Putterill J. Stevenson D. E. Kutty-Amma S. Allan A. C. (2007). Red colouration in apple fruit is due to the activity of the MYB transcription factor, MdMYB10. Plant J. 49, 414–427. 10.1111/j.1365-313X.2006.02964.x, PMID: 17181777 PMC1865000

[ref16] Feller A. Yuan L. Grotewold E. (2017). The BIF domain in plant bHLH proteins is an ACT-like domain. Plant Cell 8, 1800–1802. 10.1105/tpc.17.00356, PMID: 28747421 PMC5590504

[ref17] Finn R. D. Clements J. Eddy S. R. (2011). HMMER web server: interactive sequence similarity searching. Nucleic Acids Res. 39, W29–W37. 10.1093/nar/gkr367, PMID: 21593126 PMC3125773

[ref18] Guo W. L. Chen J. H. Li J. Huang J. Q. Wang Z. J. Lim K. J. (2020). Portal of Juglandaceae: a comprehensive platform for Juglandaceae study. Hortic. Res. 7:35. 10.1038/s41438-020-0256-x, PMID: 32194971 PMC7072074

[ref19] Heim M. A. Jakoby M. Werber M. Martin C. Weisshaar B. Bailey P. C. (2003). The basic helix-loop-helix transcription factor family in plants: a genome-wide study of protein structure and functional diversity. Mol. Biol. Evol. 20, 735–747. 10.1093/molbev/msg088, PMID: 12679534

[ref20] Hong Y. Q. Ahmad N. Tian Y. Y. Liu J. Y. Wang L. Y. Wang G.. (2019). Genome-wide identification, expression analysis, and subcellular localization of *Carthamus tinctorius* bHLH transcription factors. Int. J. Mol. Sci. 20:3044. 10.3390/ijms20123044, PMID: 31234449 PMC6627405

[ref21] Hu D. G. Sun C. H. Zhang Q. Y. An J. P. You C. X. Hao Y. J. (2016). Glucose sensor MdHXK1 phosphorylates and stabilizes MdbHLH3 to promote anthocyanin biosynthesis in apple. PLoS Genet. 12:e1006273. 10.1371/journal.pgen.1006273, PMID: 27560976 PMC4999241

[ref22] Ji W. Zhao W. Liu R. C. Jiao X. B. Han K. Yang Z. Y.. (2019). De novo assembly and transcriptome analysis of differentially expressed genes relevant to variegation in hawthorn flowers. Plant Biotechnol. Rep. 13, 579–590. 10.1007/s11816-019-00551-2

[ref23] Letunic I. Bork P. (2017). 20 years of the SMART protein domain annotation resource. Nucleic Acids Res. 46, D493–D496. 10.1093/nar/gkx922, PMID: 29040681 PMC5753352

[ref26] Li X. X. Duan X. P. Jiang H. X. Sun Y. J. Tang Y. P. Yuan Z.. (2006). Genome-wide analysis of basic/helix-loop-helix transcription factor family in rice and *Arabidopsis*. Plant Physiol. 141, 1167–1184. 10.1104/pp.106.080580, PMID: 16896230 PMC1533929

[ref27] Li Y. Z. Luo X. Wu C. Y. Cao S. Y. Zhou Y. F. Jie B.. (2018a). Comparative transcriptome analysis of genes involved in anthocyanin biosynthesis in red and green walnut (*Juglans regia* L.). Molecules 23:25. 10.3390/molecules23010025, PMID: 29271948 PMC5943948

[ref28] Li Y. Z. Shang J. H. Zhou Y. F. Wu W. J. Jie B. Wu G. L. (2018b). Determination of anthocyanins in red-fleshed walnut by ultra performance liquid chromatography-electrospray ionization tandem mass spectrometry. Food Sci. 39, 207–214. 10.7506/spkx1002-6630-201806033

[ref24] Li H. Yang Z. Zeng Q. W. Wang S. B. Luo Y. W. Huang Y.. (2020). Abnormal expression of bHLH3 disrupts a flavonoid homeostasis network, causing differences in pigment composition among mulberry fruits. Hortic. Res. 7:83. 10.1038/s41438-020-0302-8, PMID: 32528695 PMC7261776

[ref29] Liu X. Zhao C. B. Yang L. M. Zhang Y. Y. Wang Y. Fang Z.. (2020). Genome-wide identification, expression profile of the TIFY gene family in *Brassica oleracea* var. *capitata*, and their divergent response to various pathogen infections and phytohormone treatments. Genes 11:127. 10.3390/genes11020127, PMID: 31991606 PMC7073855

[ref30] Livak K. J. Schmittgen T. D. (2001). Analysis of relative gene expression data using real-time quantitative PCR and the 2^−∆∆CT^ method. Methods 25, 402–408. 10.1006/meth.2001.1262, PMID: 11846609

[ref31] Lloyd A. Brockman A. Aguirre L. Campbell A. Bean A. Cantero A.. (2017). Advances in the MYB-bHLH-WD repeat (MBW) pigment regulatory model: addition of a WRKY factor and co-option of an anthocyanin MYB for betalain regulation. Plant Cell Physiol. 58, 1431–1441. 10.1093/pcp/pcx075, PMID: 28575507 PMC5914458

[ref32] Ma D. W. Reichelt M. Yoshida K. Gershenzon J. Constabel C. P. (2018). Two R2R3-MYB proteins are broad repressors of flavonoid and phenylpropanoid metabolism in poplar. Plant J. 96, 949–965. 10.1111/tpj.14081, PMID: 30176084

[ref33] Mao K. Dong Q. L. Li C. Liu C. Liu C. H. Ma F. W. (2017). Genome wide identification and characterization of apple bHLH transcription factors and expression analysis in response to drought and salt stress. Front. Plant Sci. 8:480. 10.3389/fpls.2017.00480, PMID: 28443104 PMC5387082

[ref34] Marchler-Bauer A. Bo Y. Han L. He J. Lanczycki C. J. Lu S.. (2017). CDD/SPARCLE: functional classification of proteins via subfamily domain architectures. Nucleic Acids Res. 45, D200–D203. 10.1093/nar/gkw1129, PMID: 27899674 PMC5210587

[ref36] Matus J. T. Poupin M. J. Cañón P. Bordeu E. Alcalde J. A. Arce-Johnson P. (2010). Isolation of WDR and bHLH genes related to flavonoid synthesis in grapevine (*Vitis vinifera* L.). Plant Mol. Biol. 72, 607–620. 10.1007/s11103-010-9597-4, PMID: 20112051

[ref37] Meng J. X. Gao Y. Han M. L. Liu P. Y. Yang C. Shen T.. (2020). *In vitro* anthocyanin induction and metabolite analysis in *Malus spectabilis* leaves under low nitrogen conditions. Hortic. Plant J. 6, 284–292. 10.1016/j.hpj.2020.06.004

[ref38] Misyura M. Colasanti J. Rothstei S. J. (2013). Physiological and genetic analysis of *Arabidopsis thaliana* anthocyanin biosynthesis mutants under chronic adverse environmental conditions. J. Exp. Bot. 64, 229–240. 10.1093/jxb/ers328, PMID: 23162120 PMC3528034

[ref40] Pires N. Dolan L. (2010). Origin and diversification of basic-helix-loop-helix proteins in plants. Mol. Biol. Evol. 27, 862–874. 10.1093/molbev/msp288, PMID: 19942615 PMC2839125

[ref41] Shi Q. Q. Li X. Du J. T. Li X. G. (2019). Anthocyanin synthesis and the expression patterns of bHLH transcription factor family during development of the chinese jujube fruit (*Ziziphus jujuba* Mill.). Forests 10:346. 10.3390/f10040346

[ref42] Toledo-Ortiz G. Huq E. Quail P. H. (2003). The *Arabidopsis* basic/helix-loop-helix transcription factor family. Plant Cell 15, 1749–1770. 10.1105/tpc.013839, PMID: 12897250 PMC167167

[ref43] Wang K. J. Hao Y. B. Qi J. X. Hu X. S. (2009). Analysis of the extraction of red pellicle of walnut (*Juglans regia* L.) by ultraviolet-visible spectra and HPLC-ESI-MSn. Spectrosc. Spectr. Anal. 29, 1668–1671. 10.3964/j.issn.1000-0593(2009)06-1668-04, PMID: 19810556

[ref45] Xie X. B. Li S. Zhang R. F. Zhao J. Chen Y. C. (2012). The bHLH transcription factor MdbHLH3 promotes anthocyanin accumulation and fruit colouration in response to low temperature in apples. Plant Cell Environ. 35, 1884–1897. 10.1111/j.1365-3040.2012.02523.x, PMID: 22519753

[ref44] Xie T. Zeng L. Chen X. Rong H. Wu J. J. Batley J.. (2020). Genome-wide analysis of the lateral organ boundaries domain gene family in *Brassica napus*. Genes 11:280. 10.3390/genes11030280, PMID: 32155746 PMC7140802

[ref46] Zang Y. Jiang T. Cong Y. Zheng Z. J. Ouyang J. (2015). Molecular characterization of a recombinant *Zea mays* phenylalanine ammonia-lyase (ZmPAL2) and its application in *trans*-cinnamic acid production from L-phenylalanine. Appl. Biochem. Biotechnol. 176, 924–937. 10.1007/s12010-015-1620-4, PMID: 25947617

[ref47] Zhang J. P. Zhang W. T. Ji F. Y. Qiu J. Song X. B. Bu D. C.. (2020). A high-quality walnut genome assembly reveals extensive gene expression divergences after whole-genome duplication. Plant Biotechnol. J. 18, 1848–1850. 10.1111/pbi.13350, PMID: 32004401 PMC7415773

[ref49] Zhao M. R. Li J. Zhu L. Chang P. Li L. L. Zhang L. Y. (2019). Identification and characterization of MYB-bHLH-WD40 regulatory complex members controlling anthocyanidin biosynthesis in blueberry fruits development. Genes 10, 496–506. 10.3390/genes10070496, PMID: 31261791 PMC6678982

[ref48] Zhao H. Ren L. P. Fan X. Y. Tang K. J. Li B. (2017). Identification of putative flavonoid-biosynthetic genes through transcriptome analysis of Taihe *Toona sinensis* bud. Acta Physiol. Plant. 39:122. 10.1007/s11738-017-2422-9

[ref50] Zheng Y. W. Wu S. T. Wang R. H. Wu Y. X. Zhang W. Z. Han Y. X.. (2020). Analysis and correlationship of chemical components of various walnut (*Juglans regia* L.) cultivars. Food Meas. 14, 3605–3614. 10.1007/s11694-020-00603-0

[ref51] Zhou H. Lin-Wang K. Wang H. L. Gu C. Dare A. P. Espley R. V.. (2015). Molecular genetics of blood-fleshed peach reveals activation of anthocyanins biosynthesis by NAC transcription factors. Plant J. 82, 105–121. 10.1111/tpj.12792, PMID: 25688923

