# Supplementary material for: Genome-Wide Identification and Characterization of bHLH Transcription Factors Related to Anthocyanin Biosynthesis in Red Walnut (Juglans regia L.)
Source: Front Genet. 2021 Feb 24;12:632509. doi: 10.3389/fgene.2021.632509 (PMC7943622; doi:10.3389/fgene.2021.632509)
Supplement: Supplementary file 1 [file Data_Sheet_1.docx]

Supplementary Material

## Supplementary Figures


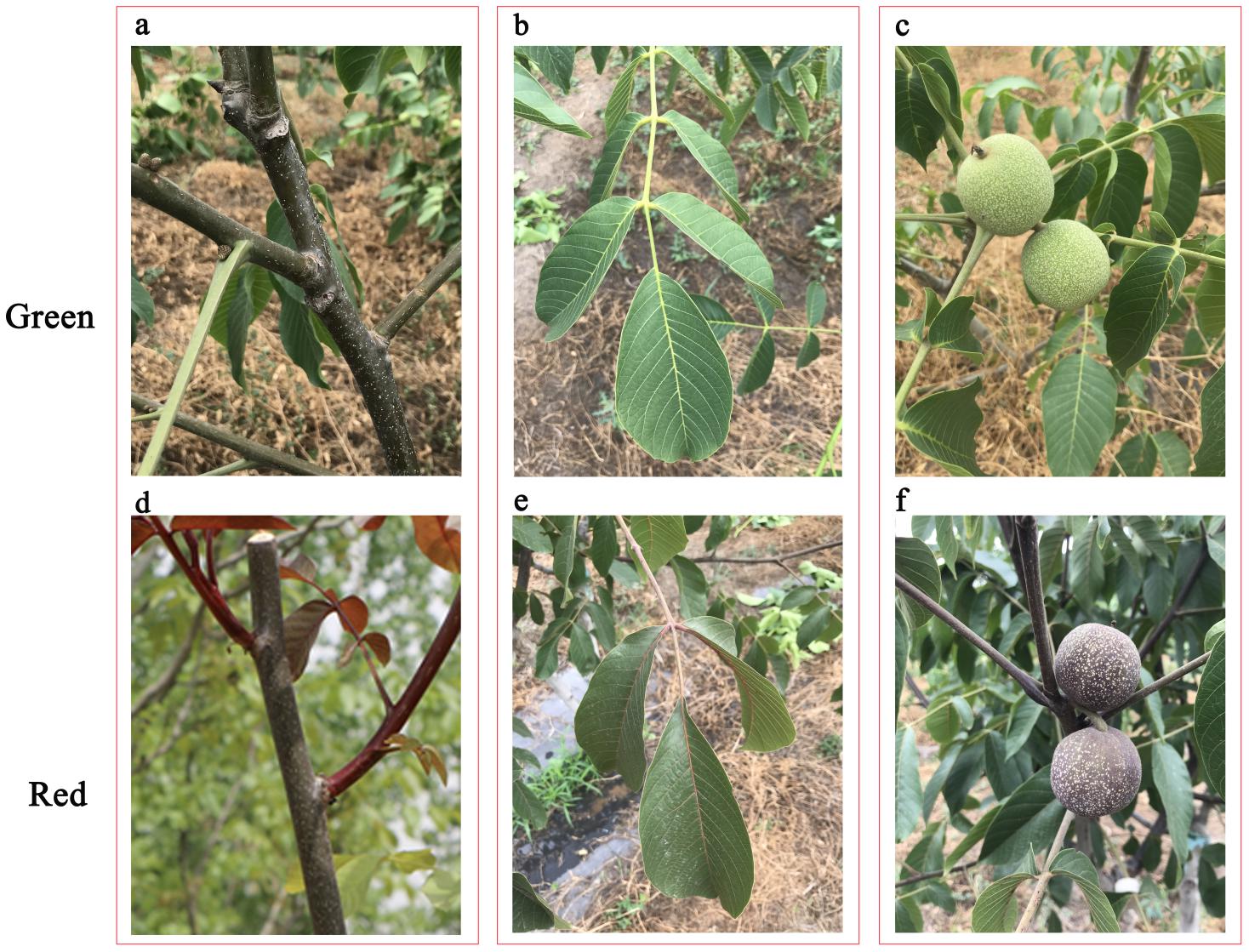


**Supplementary Figure S1.** Phenotypic comparison between common green walnut 'Zhonglin 1' and red walnut ‘RW-1’. (**a-c**) xylem, leaves and fruits of 'Zhonglin 1'. (**d-f**) xylem, leaves and fruits of ‘RW-1’.

**
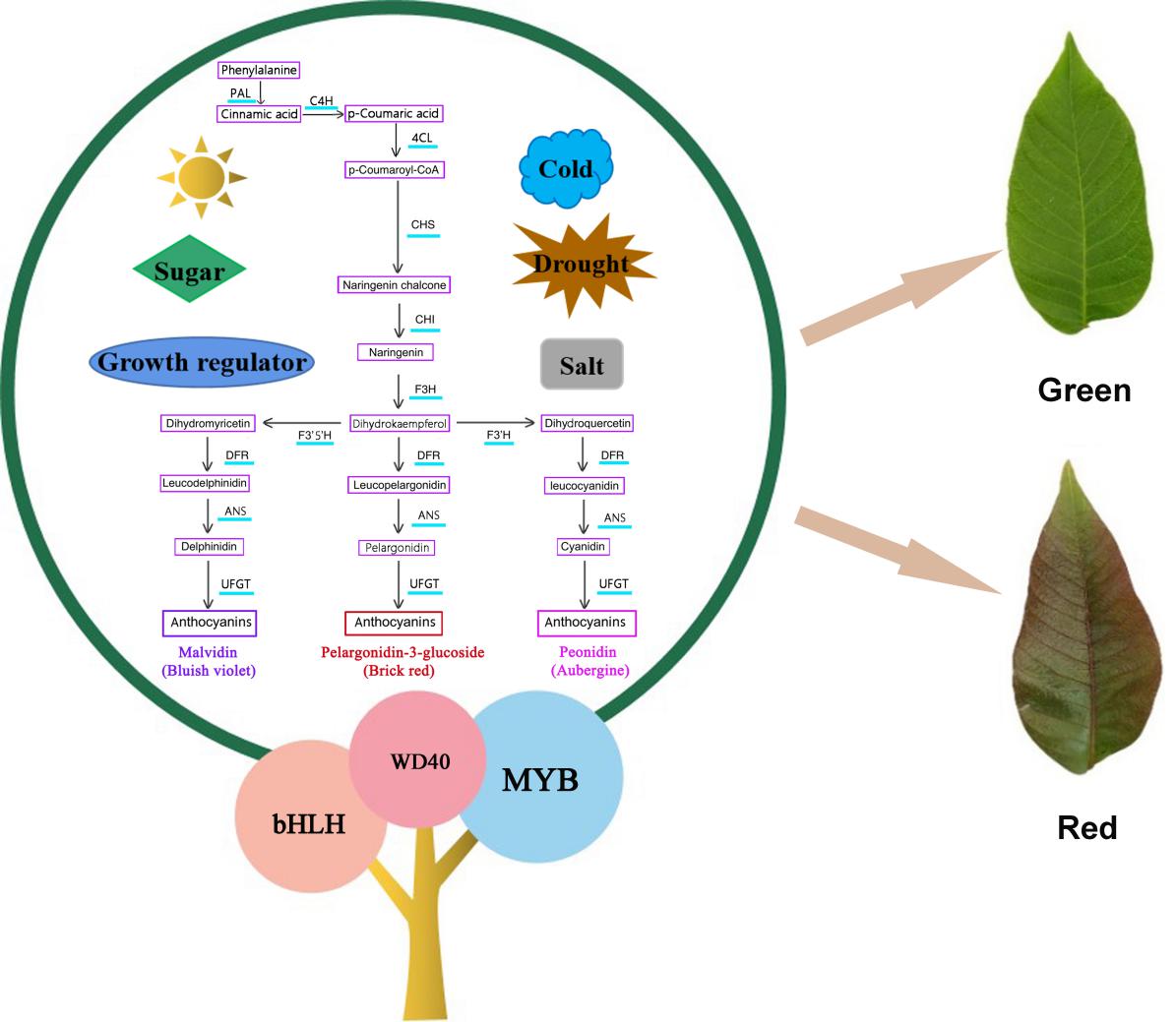
**

**Supplementary Figure S2.** Anthocyanin biosynthesis regulation model. Anthocyanin biosynthesis related genes include structural gene regulatory networks and regulatory TFs (MYB, bHLH and WD40). Light, sugar, growth regulator, cold, drought, and salt environment could also induce the coding of genes involved in anthocyanin synthesis.


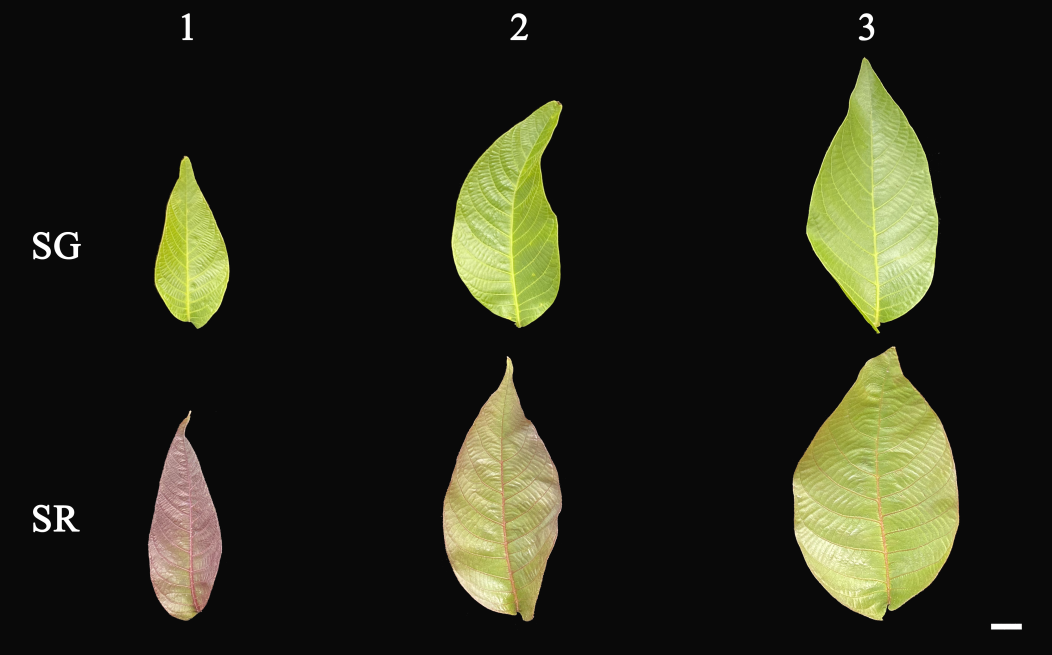


**Supplementary Figure S3.** The different phenotypes (red leaves and green leaves) of red walnut natural hybrid progeny. SG, Seedling progenies-Green leaves; SR, Seedling progenies-Red leaves. 1, the full red period (vigorous growth period of new shoots); 2, the red-green period (seed filling period); 3, the whole green period (early period of fruit ripening). The same below. Bar=1 cm.


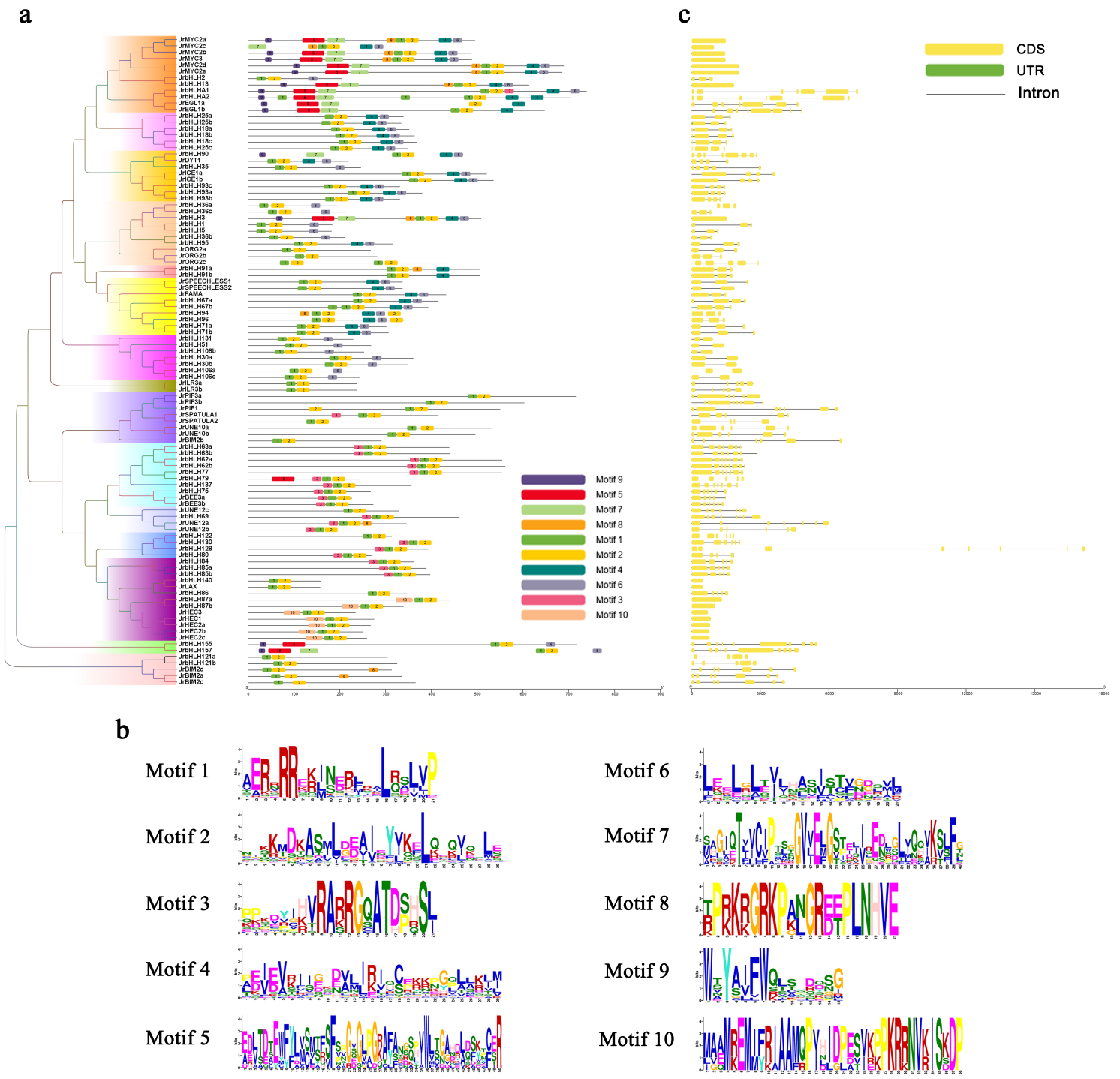


**Supplementary Figure S4.** Phylogenetic relationships, gene structure and conserved motif analysis of the walnut bHLH family. The phylogenetic tree was generated using the specifific sequences of walnut bHLH proteins (a). In the intron exon map, CDSs and UTRs are represented by yellow and green boxes respectively, and introns is represented by black lines (b). A total of 10 conserved motifs were screened and visualized with different colors (c-d) by using the MEME server.


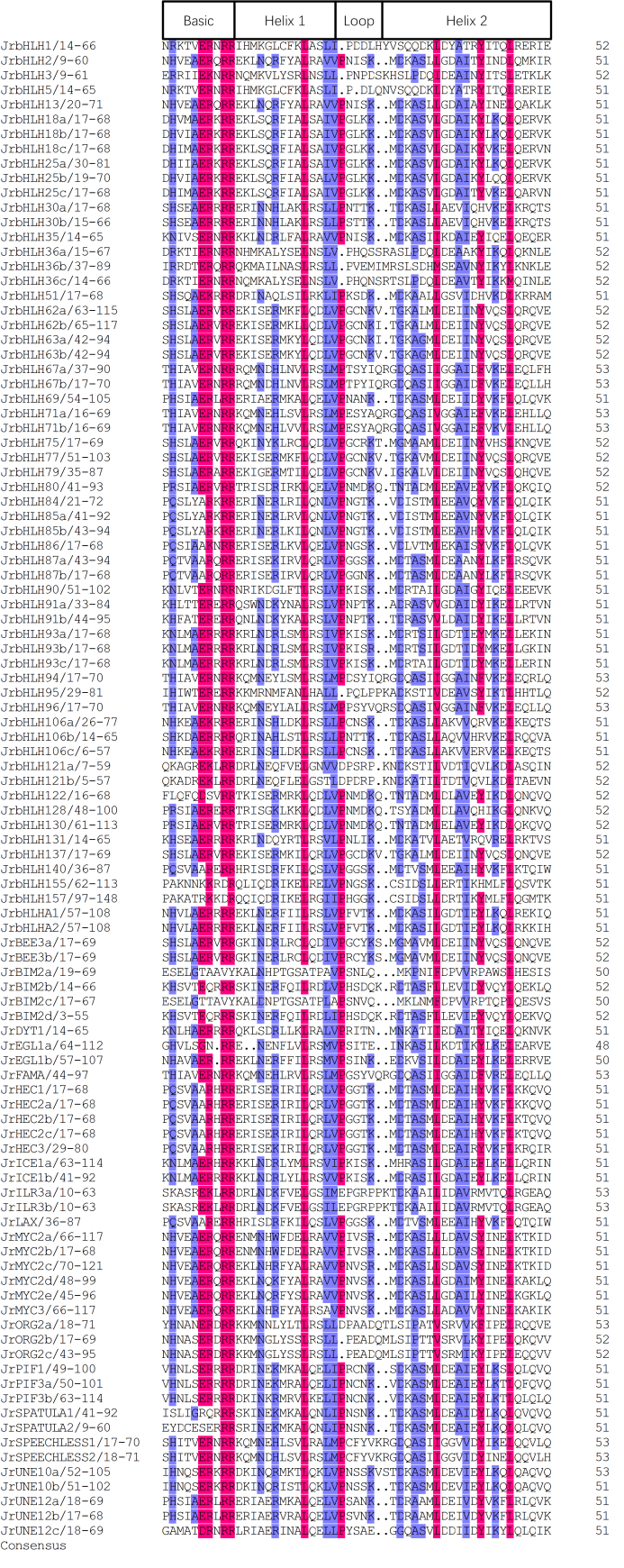


**Supplementary Figure S5.** Multiple sequence alignment of the bHLH conserved domain in walnut proteins. The purple boxes indicate 50% identity of amino acids, the red boxes indicate 75% identity of amino acids.


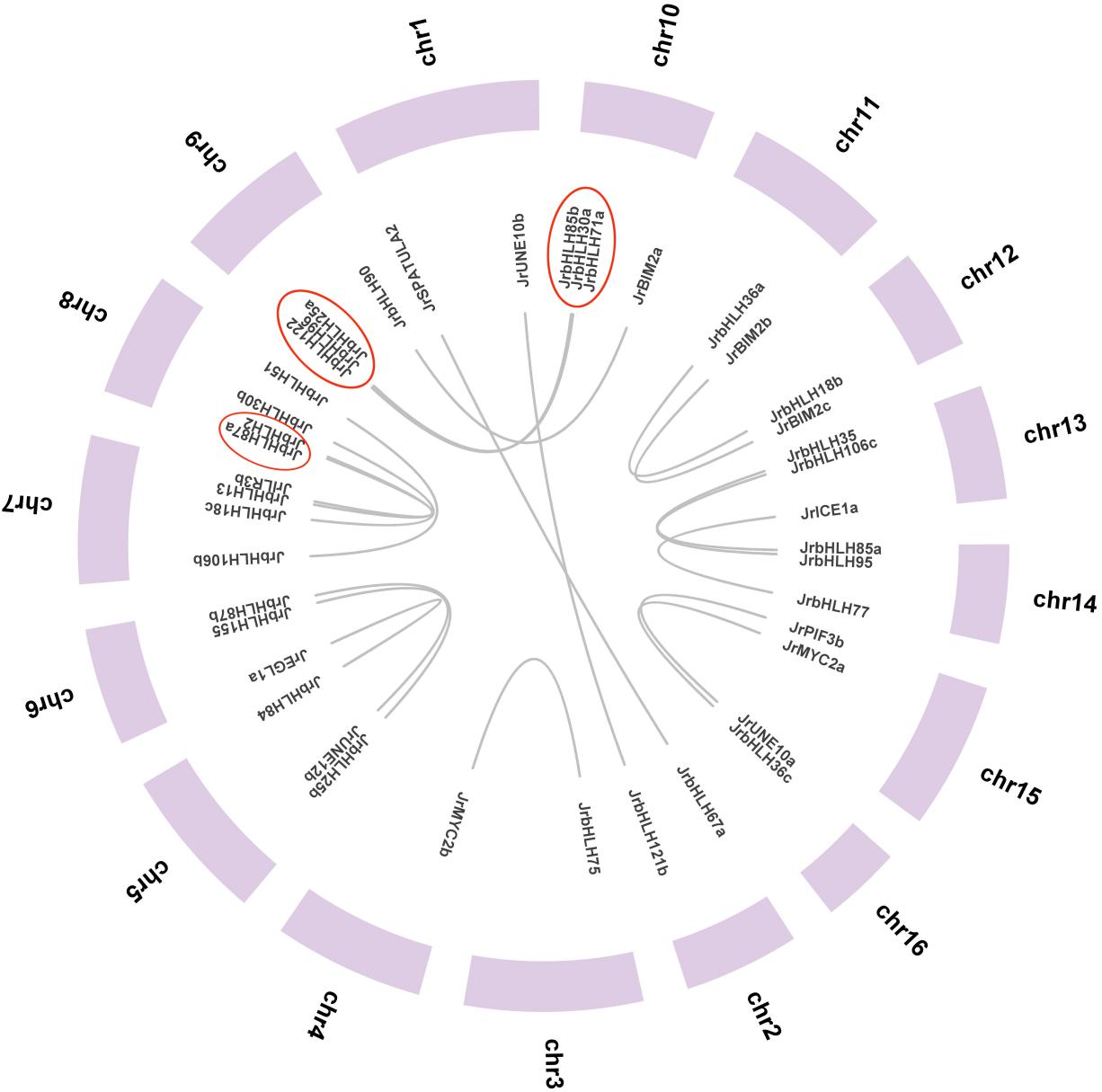


**Supplementary Figure S6.** Gene duplication events in the walnut *bHLH* gene family. The *JrbHLH* genes linked by the gray line are the segment repeats, while the *JrbHLH* genes in the red circle are the tandem repeats.


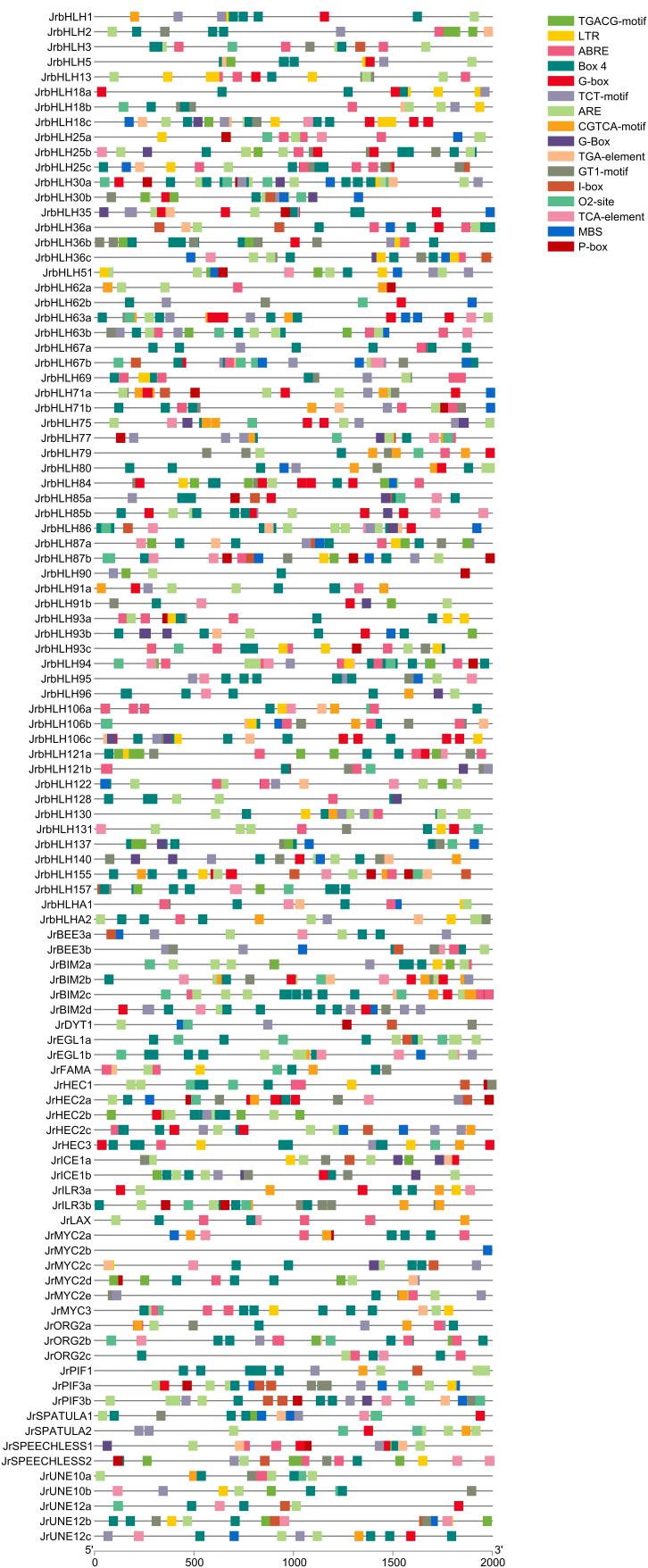


**Supplementary Figure S7.** Identified pivotal *cis*-elements in the promoters of walnut *bHLH* genes. The black line represents the upstream of the *bHLH* genes. Different colored boxes represent different *cis*-elements.


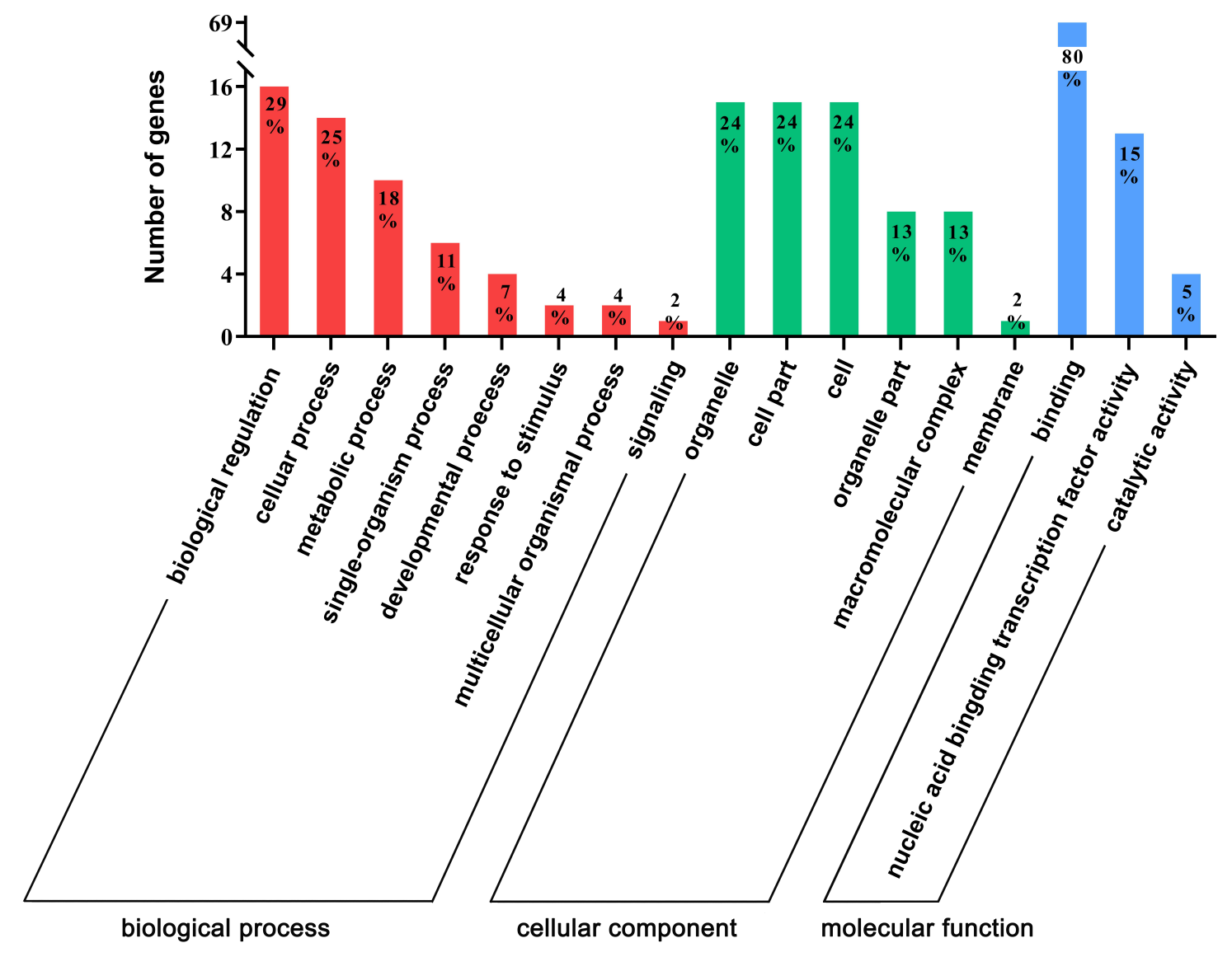


**Supplementary Figure S8.** GO annotation analysis of *JrbHLH* genes. The annotation analysis included three major categories: biological process, cellular component, and molecular function.


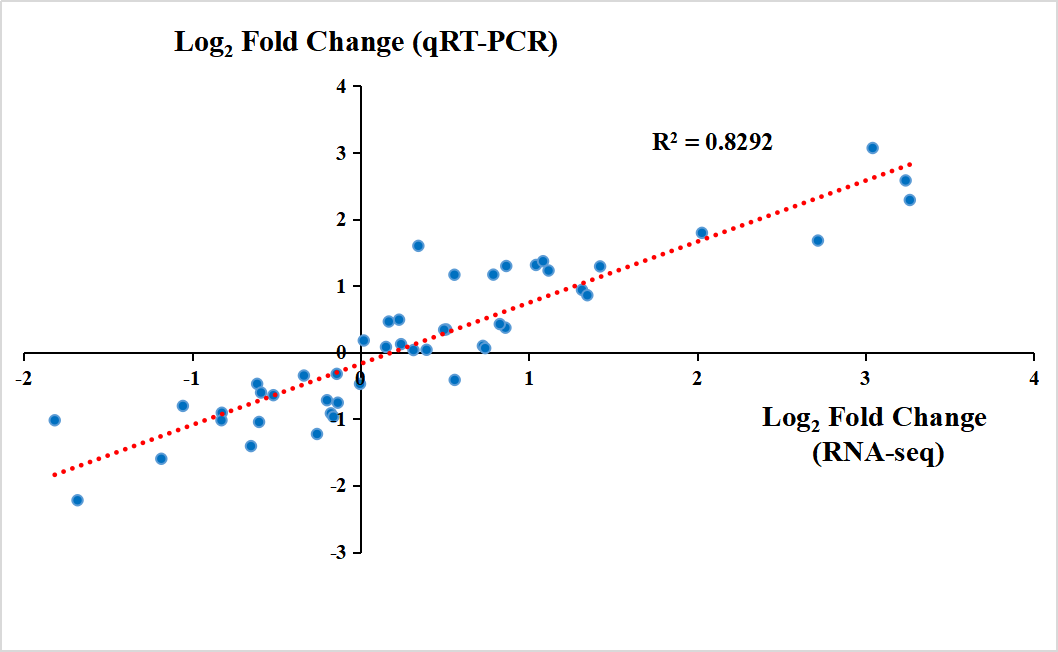


**Supplementary Figure S9.** Correlation of gene expression levels between RNA-Seq data and qRT-PCR analysis. Sixteen *JrbHLHs* in the different phenotypes (red leaves and green leaves) of red walnut natural hybrid progeny were selected and subjected to qRT-PCR analysis using the same RNA as for RNA-Seq. Both x- and y-axes are shown in Log_2_ scale.

Supplementary Tables

**Table S1.** List of primers used for qRT-PCR

| **Gene Name** | **Primer sequence (5'-3')-F** | **Primer sequence (5'-3')-R** |
| --- | --- | --- |
| *JrActin* | GCCGAACGGGAAATTGTC | AGAGATGGCTGGAAGAGG |
| *JrEGL1a* | GCCCTCAGCAGCATTGTCACC | GACCATTTGCTAGTGCCCTTCCTG |
| *JrEGL1b* | GCTGTTGGTTTGCCCGAGTCC | TTCCCTTCGCCGCCTCTCAG |
| *JrbHLHA1* | AGGAGGACGACGACGGTGAATC | CTCGGTTCGGCTGTTGTGTCTG |
| *JrbHLHA2* | TGCTGGATGGCGTCGTG | TTTGGCGGGTGAGGTTG |
| *JrbHLH36c* | CCATTGAGCGAAATAGGAGA | AACAACCTGTAAAGCCGAAC |
| *JrbHLH62a* | GAGCCTCCCAAGGACTACAT | AGCCTGGTATTGACAGAAGA |
| *JrbHLH63a* | TTTGTTTGTGGCGGAGGAGT | CATCATCAAGCACCGAGCAG |
| *JrbHLH67a* | CAGCCTCTCCGCCTCCTTGG | TCGCTTTCGCTTTCTTCGTTCCC |
| *JrbHLH69* | GCCGAACGATTACGGAGGGAG | TCCAGCATTGAAGCCTTGTCT |
| *JrbHLH71a* | CACTTCGCCTGTCCATCCTTCAC | GTGGTGAACTGCTCCTGCTATGTC |
| *JrbHLH77* | TTTCGCCGCAGACTTAC | CCTCCCTGGAATCACCC |
| *JrbHLH80* | AGCAACCACCGTCCGTCAT | GGGCTTCAATGGGTCTTCG |
| *JrbHLH87b* | TCAGAGAAGGGTCCCAGCACATC | AAGCCGCAGCCCGATAAATCATC |
| *JrbHLH96* | GCAGCAAGAACGAGGTGGAGATC | AGCGGAGCAGAGCGAGATACTC |
| *JrbHLH106a* | CCACCGCAGGATAGAGC | ATTCTTCCGCCGAGTGT |
| *JrbHLH157* | TCATTTGACCCGCTGTTAG | GATTAAAGACATCGCCTCC |

**Table S2.** List of specific primer pairs with homologous arms

| **Gene Name** | **Primer sequence (5'-3')-F** | **Primer sequence (5'-3')-R** |
| --- | --- | --- |
| *JrEGL1a* | ATACACCAAATCGACTCTAGAATGGCTAATGGCTGTCAAAC | GCCCTTGCTCACCATGGTACCACACTTACAAGCAATTTTCC |
| *JrEGL1b* | ATACACCAAATCGACTCTAGAATGGAGGGGAGAATGCTAGAAAAC | GCCCTTGCTCACCATGGTACCACACTTCCTAGTTGATCTCTGG |
| *JrbHLHA1* | ATACACCAAATCGACTCTAGAATGGCTGCACCGCCGAG | GCCCTTGCTCACCATGGTACCAGAGTCTGTGTGGGGGATG |
| *JrbHLHA2* | ATACACCAAATCGACTCTAGAATGGCTGCACCGCCAA | GCCCTTGCTCACCATGGTACCAGAGTCATTGTGGGGTATG |

Table S3. List of walnut bHLH protein family genes and their physiochemical properties.

| **Gene name** | **Protein ID** | **Gene ID** | **CDS ID** | **Amino Acid**  **(aa)** | **CDS**  **(bp)** | **pI** | **Molecular weight (kDa)** | **Subcellular location** |
| --- | --- | --- | --- | --- | --- | --- | --- | --- |
| *JrbHLH1* | XP_018831547.1 | NW_017443629.1:508469-511180 | XM_018976002.1 | 182 | 549 | 9.56 | 20.68 | Nucleus |
| *JrbHLH2* | XP_018842915.1 | NW_017389429.1:5295-6503 | XM_018987370.1 | 204 | 615 | 6.32 | 23.12 | Nucleus |
| *JrbHLH3* | XP_018827271.1 | NW_017443590.1:460181-462642 | XM_018971726.1 | 507 | 1,524 | 5.76 | 56.02 | Nucleus |
| *JrbHLH5* | XP_018817318.1 | NW_017443031.1:1342242-1343466 | XM_018961773.1 | 181 | 546 | 9.64 | 20.49 | Nucleus |
| *JrbHLH13* | XP_018818377.1 | NW_017443224.1:c254993-257908 | XM_018962832.1 | 612 | 1,839 | 6.32 | 67.96 | Nucleus |
| *JrbHLH18a* | XP_018813765.1 | NW_017442762.1:73581-76022 | XM_018958220.1 | 350 | 1,053 | 6.17 | 38.61 | Nucleus |
| *JrbHLH18b* | XP_018817560.1 | NW_017443088.1:1989-4984 | XM_018962015.1 | 362 | 1,089 | 7.09 | 40.49 | Nucleus |
| *JrbHLH18c* | XP_018813188.1 | NW_017442685.1:c17838-20285 | XM_018957643.1 | 366 | 1,101 | 5.97 | 40.79 | Nucleus |
| *JrbHLH25a* | XP_018844450.1 | NW_017389528.1:c45558-48372 | XM_018988905.1 | 338 | 1,017 | 8.58 | 37.90 | Nucleus |
| *JrbHLH25b* | XP_018826860.1 | NW_017443584.1:114027-116075 | XM_018971315.1 | 333 | 1,002 | 7.16 | 37.74 | Nucleus |
| *JrbHLH25c* | XP_018813766.1 | NW_017442762.1:52486-54171 | XM_018958221.1 | 348 | 1,047 | 6.23 | 39.05 | Nucleus |
| *JrbHLH30a* | XP_018836098.1 | NW_017388825.1:c139299-141949 | XM_018980553.1 | 359 | 1,080 | 6.17 | 39.79 | Nucleus |
| *JrbHLH30b* | XP_018823686.1 | NW_017443545.1:c224421-226967 | XM_018968141.1 | 348 | 1,047 | 5.96 | 38.57 | Nucleus |
| *JrbHLH35* | XP_018836607.1 | NW_017389065.1:51230-54665 | XM_018981062.1 | 245 | 738 | 4.97 | 27.84 | Nucleus |
| *JrbHLH36a* | XP_018852453.1 | NW_017389987.1:30733-31736 | XM_018996908.1 | 193 | 582 | 7.04 | 21.51 | Mitochondrial |
| *JrbHLH36b* | XP_018830661.1 | NW_017443621.1:c1540014-1541198 | XM_018975116.1 | 211 | 636 | 8.99 | 23.88 | Nucleus |
| *JrbHLH36c* | XP_018852439.1 | NW_017389987.1:121808-123074 | XM_018996894.1 | 209 | 630 | 5.63 | 23.41 | Nucleus |
| *JrbHLH51* | XP_018846326.1 | NW_017389644.1:26720-28562 | XM_018990781.1 | 267 | 804 | 8.82 | 29.61 | Nucleus |
| *JrbHLH62a* | XP_018827778.1 | NW_017443591.1:378732-381777 | XM_018972233.1 | 553 | 1,662 | 6.38 | 60.35 | Nucleus |
| *JrbHLH62b* | XP_018844170.1 | NW_017389499.1:244634-247893 | XM_018988625.1 | 560 | 1,683 | 6.09 | 60.95 | Nucleus |
| *JrbHLH63a* | XP_018818185.1 | NW_017443173.1:c2860-5594 | XM_018962640.1 | 438 | 1,317 | 5.75 | 47.94 | Nucleus |
| *JrbHLH63b* | XP_018851563.1 | NW_017389935.1:c39509-42874 | XM_018996018.1 | 440 | 1,323 | 5.80 | 48.65 | Nucleus |
| *JrbHLH67a* | XP_018822760.1 | NW_017443536.1c498969-501479 | XM_018967215.1 | 412 | 1,239 | 5.67 | 46.80 | Nucleus |
| *JrbHLH67b* | XP_018808973.1 | NW_017441213.1:283813-286351 | XM_018953428.1 | 392 | 1,179 | 6.00 | 44.66 | Nucleus |
| *JrbHLH69* | XP_018833391.1 | NW_017388808.1:131182-134821 | XM_018977846.1 | 460 | 1,383 | 5.94 | 48.66 | Nucleus |
| *JrbHLH71a* | XP_018808815.1 | NW_017441128.1:127250-130121 | XM_018953270.1 | 300 | 903 | 8.30 | 33.99 | Nucleus |
| *JrbHLH71b* | XP_018812455.1 | NW_017442556.1:54096-57465 | XM_018956910.1 | 305 | 918 | 9.33 | 34.44 | Nucleus |
| *JrbHLH75* | XP_018816944.1 | NW_017443009.1:2908947-2910807 | XM_018961399.1 | 266 | 801 | 5.90 | 30.11 | Nucleus |
| *JrbHLH77* | XP_018828245.1 | NW_017443596.1:c450176-453508 | XM_018972700.1 | 553 | 1,662 | 6.15 | 60.40 | Nucleus |
| *JrbHLH79* | XP_018819056.1 | NW_017443271.1:c400012-403106 | XM_018963511.1 | 242 | 729 | 6.46 | 26.59 | Nucleus |
| *JrbHLH80* | XP_018833612.1 | NW_017388951.1:c445602-449613 | XM_018978067.1 | 268 | 807 | 5.03 | 29.72 | Nucleus |
| *JrbHLH84* | XP_018837996.1 | NW_017389164.1:22569-24534 | XM_018982451.1 | 360 | 1,083 | 5.02 | 39.60 | Nucleus |
| *JrbHLH85a* | XP_018859013.1 | NW_017437648.1:c60173-61819 | XM_019003468.1 | 388 | 1,167 | 4.91 | 43.03 | Nucleus |
| *JrbHLH85b* | XP_018810275.1 | NW_017441917.1:c96677-98320 | XM_018954730.1 | 396 | 1,191 | 5.19 | 43.71 | Nucleus |
| *JrbHLH86* | XP_018816619.1 | NW_017443009.1:2331953-2333532 | XM_018961074.1 | 347 | 1,044 | 5.96 | 38.50 | Nucleus |
| *JrbHLH87a* | XP_018843297.1 | NW_017389449.1:c515358-516951 | XM_018987752.1 | 438 | 1,317 | 6.78 | 48.43 | Nucleus |
| *JrbHLH87b* | XP_018808805.1 | NW_017441114.1:9940-14514 | XM_018953260.1 | 338 | 1,017 | 7.60 | 37.34 | Nucleus |
| *JrbHLH90* | XP_018815047.1 | NW_017442853.1:c254812-258315 | XM_018959502.1 | 494 | 1,485 | 6.01 | 55.39 | Nucleus |
| *JrbHLH91a* | XP_018825414.1 | NW_017443569.1:c15483-59912 | XM_018969869.1 | 504 | 1,515 | 5.38 | 56.13 | Nucleus |
| *JrbHLH91b* | XP_018825412.1 | NW_017443569.1:c15483-59912 | XM_018969867.1 | 505 | 1,518 | 5.44 | 56.34 | Nucleus |
| *JrbHLH93a* | XP_018821081.1 | NW_017443495.1:44475-45935 | XM_018965536.1 | 379 | 1,140 | 5.02 | 42.73 | Nucleus |
| *JrbHLH93b* | XP_018848702.1 | NW_017389791.1:c1309936-1311744 | XM_018993157.1 | 330 | 993 | 4.79 | 37.11 | Nucleus |
| *JrbHLH93c* | XP_018813428.1 | NW_017442725.1:c66977-68793 | XM_018957883.1 | 330 | 993 | 5.04 | 37.35 | Nucleus |
| *JrbHLH94* | XP_018841767.1 | NW_017389361.1:c979617-981214 | XM_018986222.1 | 339 | 1,020 | 5.12 | 38.28 | Nucleus |
| *JrbHLH95* | XP_018831533.1 | NW_017443629.1:c2679295-2681625 | XM_018975988.1 | 314 | 945 | 7.76 | 34.05 | Nucleus |
| *JrbHLH96* | XP_018833943.1 | NW_017388955.1:c1081804-1083695 | XM_018978398.1 | 341 | 1,026 | 6.23 | 38.52 | Nucleus |
| *JrbHLH106a* | XP_018810563.1 | NW_017442006.1:157628-160678 | XM_018955018.1 | 254 | 765 | 7.12 | 28.44 | Nucleus |
| *JrbHLH106b* | XP_018858554.1 | NW_017437336.1:36320-37625 | XM_019003009.1 | 252 | 759 | 9.21 | 27.23 | Nucleus |
| *JrbHLH106c* | XP_018814123.1 | NW_017442800.1:14504-16671 | XM_018958578.1 | 242 | 729 | 7.66 | 27.16 | Nucleus |
| *JrbHLH121a* | XP_018841437.1 | NW_017389360.1:c412634-416730 | XM_018985892.1 | 303 | 912 | 6.16 | 33.68 | Nucleus |
| *JrbHLH121b* | XP_018846432.1 | NW_017389654.1:11731-15231 | XM_018990887.1 | 324 | 975 | 6.55 | 35.87 | Nucleus |
| *JrbHLH122* | XP_018851501.1 | NW_017389931.1:580227-583318 | XM_018995956.1 | 312 | 939 | 5.22 | 33.83 | Nucleus |
| *JrbHLH128* | XP_018815769.1 | NW_017442877.1:c1287304-1304908 | XM_018960224.1 | 392 | 1,179 | 9.24 | 42.03 | Nucleus |
| *JrbHLH130* | XP_018830949.1 | NW_017443622.1:663481-666831 | XM_018975404.1 | 414 | 1245 | 6.84 | 46.10 | Nucleus |
| *JrbHLH131* | XP_018820968.1 | NW_017443485.1:289214-290224 | XM_018965423.1 | 228 | 687 | 10.46 | 26.11 | Mitochondrial |
| *JrbHLH137* | XP_018832505.1 | NW_017388807.1:c912666-915629 | XM_018976960.1 | 355 | 1,068 | 8.44 | 39.81 | Nucleus |
| *JrbHLH140* | XP_018859333.1 | NW_017437856.1:3599-4189 | XM_019003788.1 | 158 | 477 | 6.91 | 17.87 | Nucleus |
| *JrbHLH155* | XP_018827228.1 | NW_017443590.1:c736184-743013 | XM_018971683.1 | 717 | 2,154 | 5.86 | 78.90 | Nucleus |
| *JrbHLH157* | XP_018858098.1 | NW_017437045.1:c10770-16293 | XM_019002553.1 | 842 | 2,529 | 4.82 | 92.50 | Nucleus |
| *JrbHLHA1* | XP_018814174.1 | NW_017442805.1:115556-123235 | XM_018958629.1 | 738 | 2,217 | 5.24 | 81.99 | Nucleus |
| *JrbHLHA2* | XP_018812928.1 | NW_017442642.1:42146-70828 | XM_018957383.1 | 702 | 2,109 | 5.58 | 78.49 | Nucleus |
| *JrBEE3a* | XP_018806041.1 | NW_017439360.1:45158-46994 | XM_018950496.1 | 225 | 678 | 8.63 | 25.32 | Nucleus |
| *JrBEE3b* | XP_018830355.1 | NW_017388910.1:c52266..53880 | XM_018974810.1 | 272 | 819 | 7.02 | 30.79 | Nucleus |
| *JrBIM2a* | XP_018846945.1 | NW_017389678.1:14285-19041 | XM_018991400.1 | 335 | 1,008 | 5.45 | 36.91 | Nucleus |
| *JrBIM2b* | XP_018860393.1 | NW_017438565.1:20111-27322 | XM_019004848.1 | 290 | 873 | 5.53 | 32.57 | Nucleus |
| *JrBIM2c* | XP_018816956.1 | NW_017443009.1:c1078906-1083834 | XM_018961411.1 | 364 | 1,095 | 5.45 | 40.94 | Nucleus |
| *JrBIM2d* | XP_018810846.1 | NW_017442092.1:11880-17299 | XM_018955301.1 | 312 | 939 | 7.10 | 34.66 | Nucleus |
| *JrDYT1* | XP_018818442.1 | NW_017443250.1:68839-70499 | XM_018962897.1 | 218 | 657 | 6.04 | 24.66 | Nucleus |
| *JrEGL1a* | XP_018807981.1 | NW_017440501.1:c29383-34641 | XM_018952436.1 | 656 | 1,971 | 5.00 | 73.77 | Nucleus |
| *JrEGL1b* | XP_018852488.1 | NW_017389990.1:382073-386768 | XM_018996943.1 | 646 | 1,941 | 5.12 | 72.83 | Nucleus |
| *JrFAMA* | XP_018860283.1 | NW_017388876.1:36165-38558 | XM_019004738.1 | 431 | 1,296 | 5.38 | 48.90 | Nucleus |
| *JrHEC1* | XP_018844966.1 | NW_017389563.1:c104236-105302 | XM_018989421.1 | 274 | 825 | 8.83 | 30.40 | Nucleus |
| *JrHEC2a* | XP_018813738.1 | NW_017442759.1:c123412-124415 | XM_018958193.1 | 266 | 801 | 9.01 | 29.30 | Nucleus |
| *JrHEC2b* | XP_018856470.1 | NW_017436177.1:c89327-90361 | XM_019000925.1 | 250 | 753 | 6.91 | 27.75 | Nucleus |
| *JrHEC2c* | XP_018860678.1 | NW_017438739.1:23014-25187 | XM_019005133.1 | 258 | 777 | 9.48 | 28.52 | Nucleus |
| *JrHEC3* | XP_018859087.1 | NW_017437675.1:50010-51101 | XM_019003542.1 | 233 | 702 | 6.23 | 26.22 | Nucleus |
| *JrICE1a* | XP_018832724.1 | NW_017388807.1:1019413-1022844 | XM_018977179.1 | 520 | 1,563 | 6.05 | 57.39 | Nucleus |
| *JrICE1b* | XP_018836604.1 | NW_017388828.1:c222945-226349 | XM_018981059.1 | 534 | 1,605 | 5.36 | 57.84 | Nucleus |
| *JrILR3a* | XP_018852414.1 | NW_017388857.1:907583-910707 | XM_018996869.1 | 236 | 711 | 6.63 | 25.81 | Nucleus |
| *JrILR3b* | XP_018823925.1 | NW_017388898.1:1119128-1121739 | XM_018968380.1 | 236 | 711 | 6.64 | 25.87 | Nucleus |
| *JrLAX* | XP_018826182.1 | NW_017443575.1:c685352-685822 | XM_018970637.1 | 156 | 471 | 6.91 | 17.59 | Nucleus |
| *JrMYC2a* | XP_018860395.1 | NW_017388876.1:201470-203219 | XM_019004850.1 | 494 | 1,485 | 5.61 | 55.62 | Nucleus |
| *JrMYC2b* | XP_018815973.1 | NW_017442914.1:c6839-8074 | XM_018960428.1 | 322 | 969 | 5.91 | 36.23 | Nucleus |
| *JrMYC2c* | XP_018805267.1 | NW_017438865.1:186963-189077 | XM_018949722.1 | 484 | 1,455 | 6.00 | 53.83 | Nucleus |
| *JrMYC2d* | XP_018811463.1 | NW_017442309.1:59141-61805 | XM_018955918.1 | 688 | 2,067 | 5.41 | 75.50 | Nucleus |
| *JrMYC2e* | XP_018817566.1 | NW_017443092.1:c54450-57112 | XM_018962021.1 | 684 | 2,055 | 5.56 | 74.85 | Nucleus |
| *JrMYC3* | XP_018814191.1 | NW_017442807.1:161324-163078 | XM_018958646.1 | 487 | 1,464 | 8.09 | 54.50 | Nucleus |
| *JrORG2a* | XP_018851306.1 | NW_017388857.1:260098-262400 | XM_018995761.1 | 266 | 801 | 7.14 | 30.38 | Nucleus |
| *JrORG2b* | XP_018848398.1 | NW_017389776.1:c115368-117013 | XM_018992853.1 | 280 | 843 | 5.78 | 31.61 | Nucleus |
| *JrORG2c* | XP_018826176.1 | NW_017388899.1:c84738-85998 | XM_018970631.1 | 435 | 1,308 | 6.03 | 48.37 | Nucleus |
| *JrPIF1* | XP_018820337.1 | NW_017443356.1:27724-36836 | XM_018964792.1 | 549 | 1,650 | 6.26 | 60.32 | Nucleus |
| *JrPIF3a* | XP_018856447.1 | NW_017436176.1:c3332-38701 | XM_019000902.1 | 714 | 2,145 | 6.27 | 76.59 | Nucleus |
| *JrPIF3b* | XP_018843145.1 | NW_017389441.1:c583963-589402 | XM_018987600.1 | 602 | 1,809 | 8.70 | 65.30 | Nucleus |
| *JrSPATULA1* | XP_018822301.1 | NW_017443528.1:188899..193341 | XM_018966756.1 | 414 | 1,245 | 6.28 | 45.23 | Nucleus |
| *JrSPATULA2* | XP_018844989.1 | NW_017389563.1:c170479-174682 | XM_018989444.1 | 281 | 846 | 5.69 | 30.71 | Nucleus |
| *JrSPEECHLESS1* | XP_018812131.1 | NW_017442460.1:c32978-36214 | XM_018956586.1 | 336 | 1,011 | 5.58 | 37.17 | Nucleus |
| *JrSPEECHLESS2* | XP_018852097.1 | NW_017389966.1:c31253-34455 | XM_018996552.1 | 336 | 1,011 | 6.47 | 37.20 | Nucleus |
| *JrUNE10a* | XP_018853938.1 | NW_017388861.1:c1108142-1113029 | XM_018998393.1 | 530 | 1,593 | 8.74 | 57.44 | Nucleus |
| *JrUNE10b* | XP_018820122.1 | NW_017388893.1:2583711-2588203 | XM_018964577.1 | 495 | 1,488 | 6.49 | 53.21 | Nucleus |
| *JrUNE12a* | XP_018830228.1 | NW_017443616.1:c118562-125540 | XM_018974683.1 | 345 | 1,038 | 5.38 | 36.70 | Nucleus |
| *JrUNE12b* | XP_018812362.1 | NW_017442542.1:13179-18537 | XM_018956817.1 | 294 | 885 | 5.90 | 31.39 | Nucleus |
| *JrUNE12c* | XP_018805725.1 | NW_017439142.1:73487-76770 | XM_018950180.1 | 328 | 987 | 4.78 | 36.17 | Nucleus |

**Table S4.** Functionally annotated cis-elements identified in the promoters of walnut *bHLH* genes

| **Categories** | **Cis-Element** | **Functions of *Cis*-Elements** |
| --- | --- | --- |
| Light-response elements | Box 4 | part of a conserved DNA module involved in light responsiveness |
|  | G-box | cis-acting regulatory element involved in light responsiveness |
|  | GT1-motif | light responsive element |
|  | TCT-motif | part of a light responsive element |
|  | I-box | part of a light responsive element |
| Stress-response elements | ARE | cis-acting regulatory element essential for the anaerobic induction |
|  | MBS | MYB binding site involved in drought-inducibility |
|  | LTR | cis-acting element involved in low-temperature responsiveness |
| Hormone-response elements | ABRE | cis-acting element involved in the abscisic acid responsiveness |
|  | CGTCA-motif | cis-acting regulatory element involved in the MeJA-responsiveness |
|  | TGACG-motif | cis-acting regulatory element involved in the MeJA-responsiveness |
|  | O2-site | cis-acting regulatory element involved in zein metabolism regulation |
|  | TCA-element | cis-acting element involved in salicylic acid responsiveness |
|  | TGA-element | auxin-responsive element |
|  | P-box | gibberellin-responsive element |

**Table S5.** Statistics of sequencing data results

| **Samples** | **Total reads** | **Clean reads** | **Clean bases** | **GC Content (%)** | **Q30 (%)** | **Comparison efficiency (%)** |
| --- | --- | --- | --- | --- | --- | --- |
| Jr-SG-1-1 | 48,438,004 | 24,219,002 | 7.24 G | 47.46% | 95.80% | 94.11% |
| Jr-SG-1-2 | 47,508,468 | 23,754,234 | 7.10 G | 47.48% | 95.46% | 93.07% |
| Jr-SG-1-3 | 51,388,058 | 25,694,029 | 7.67 G | 47.45% | 95.67% | 92.73% |
| Jr-SG-2-1 | 57,833,170 | 28,916,585 | 8.64 G | 47.37% | 95.48% | 93.57% |
| Jr-SG-2-2 | 46,162,588 | 23,081,294 | 6.90 G | 47.62% | 95.40% | 92.23% |
| Jr-SG-2-3 | 50,026,542 | 25,013,271 | 7.48 G | 47.45% | 95.43% | 93.30% |
| Jr-SG-3-1 | 44,386,476 | 22,193,238 | 6.64 G | 47.50% | 95.23% | 92.38% |
| Jr-SG-3-2 | 44,956,344 | 22,478,172 | 6.72 G | 47.78% | 95.27% | 92.89% |
| Jr-SG-3-3 | 47,011,944 | 23,505,972 | 7.02 G | 47.45% | 95.44% | 94.00% |
| Jr-SR-1-1 | 62,191,784 | 31,095,892 | 9.30 G | 47.45% | 95.53% | 95.01% |
| Jr-SR-1-2 | 53,125,670 | 26,562,835 | 7.95 G | 47.49% | 95.84% | 95.27% |
| Jr-SR-1-3 | 48,730,032 | 24,365,016 | 7.28 G | 47.50% | 95.90% | 93.63% |
| Jr-SR-2-1 | 50,195,350 | 25,097,675 | 7.50 G | 47.60% | 95.58% | 93.32% |
| Jr-SR-2-2 | 43,291,604 | 21,645,802 | 6.47 G | 47.60% | 95.50% | 94.64% |
| Jr-SR-2-3 | 48,733,196 | 24,366,598 | 7.29 G | 47.53% | 95.22% | 93.68% |
| Jr-SR-3-1 | 49,103,064 | 24,551,532 | 7.34 G | 47.16% | 95.34% | 94.39% |
| Jr-SR-3-2 | 55,305,866 | 27,652,933 | 8.26 G | 47.62% | 95.58% | 93.06% |
| Jr-SR-3-3 | 48,342,068 | 24,171,034 | 7.21 G | 47.69% | 95.75% | 93.20% |
